# Amazonian useful plants described in the book “Le Pays des Amazones” (1885) of the Brazilian propagandist Baron de Santa-Anna Nery: a historical and ethnobotanical perspective

**DOI:** 10.1186/s13002-024-00663-2

**Published:** 2024-02-26

**Authors:** Lucas N. R. Silva, Elaine C. P. Oliveira, Leopoldo C. Baratto

**Affiliations:** 1 Laboratory of Medicinal Plant Biotechnology, Post-Graduate Program in Biosciences, Universidade Federal do Oeste do Pará (UFOPA), Santarém, Pará (PA) Brazil; 2 Laboratory of Applied Pharmacognosy, Faculty of Pharmacy, Universidade Federal do Rio de Janeiro (UFRJ), Rio de Janeiro, Rio de Janeiro (RJ) Brazil

**Keywords:** Historical ethnobotany, Historical records, Natural history, Biodiversity, Amazonian plants, Brazilian medicinal plants

## Abstract

**Background:**

Frederico José de Santa-Anna Nery (1848–1901) was a Brazilian Baron who referred to himself as a "volunteer propagandist" for Brazil in Europe, serving as an immigration agent to publicize the living conditions in the Amazon region, advocating for its development and modernization at the end of the nineteenth century. Santa-Anna Nery’s most famous book is "Le Pays des Amazones" (The Lands of the Amazons), first published in 1885, which the author dedicated a chapter to introduce and report on the Amazonian useful plant species and its relationship with humans. The aim of this work is to understand the historical context and ethnobotanical value of the plant species in the Brazilian Amazon at the end of the nineteenth century through an analysis of the book “Le Pays des Amazones” (1885) by Baron de Santa-Anna Nery, as well as to bring to light the historical importance of this very influential propagandist, who has been forgotten nowadays.

**Methods:**

The original book “Le Pays des Amazones” (1885), as well as the original 3rd edition and its translated version into Portuguese, was carefully analyzed and all information about plants was systematized, with botanical names being updated. Finally, using the scientific name of medicinal plants alone or in combination with their traditional use, a search was carried out in databases in order to indicate current pharmacological studies that provide evidence about the described traditional uses.

**Results:**

A total of 156 plant species were identified in the book, although 132 species had their scientific names updated. These species belong to 45 different families, with Fabaceae and Arecaceae the most represented, and 109 plants are Brazilian native. Considering only the 36 medicinal plants, the main medicinal indications reported were astringent, purgative/laxative, stimulant and tonic, vermifuge, febrifuge, sudorific, emetic, diuretic and antidysenteric. Regarding other useful plants (non-medicinal), 97 species were cited for food, constructions and buildings, spices and condiments, ornaments and objects, carpentry, textile fibers, gums, oils, balms and essences, pigments and tanning, hunting and fishing.

**Conclusions:**

When the book “Le Pays des Amazones” is analyzed from a timeless perspective, with a particular focus on historical ethnobotany, it is possible to observe the economic, social, and political importance of many useful plants for the Amazon at the end of the nineteenth century and how the relationship between local people, indigenous communities, and immigrants was established with plant biodiversity.

## Background

### The naturalists in South America

During the nineteenth century in Brazil, significant scientific expeditions were conducted by various naturalists with the purpose of cataloging the still undescribed biodiversity, mapping territories, promoting agriculture, exploring native natural products, and developing foreign trade [1, 2]. It is important to have clear that the South American biodiversity was unknown under a perspective of European colonizers but not to the indigenous peoples, which interacted with nature since millennia and knew properly the medicinal, edible, and other potentials of plant species, as well as the localities to collect the plants and how to cultivate some species. Unfortunately, indigenous peoples are still eclipsed in the historiography of the production and circulation of knowledge and technologies [3]. The naturalists, mostly Europeans, made valuable written records of the traditional use of plants in South America, with notable figures coming to Brazil such as Maximilian zu Wied-Neuwied (1815–1817) [4], Carl F. P. von Martius (1817–1820) [5], Auguste de Saint-Hilaire (1816–1822) [6], George Gardner (1836–1841) [7], Alfred Russel Wallace (1848–1852) [8], Henry Walter Bates (1848–1859) [9, 10], Richard Spruce (1849–1864) [11], among others [1, 12].

Many other naturalists were inventorying plant species in South America since the sixteenth century, such as Antonio Pigafetta (1520) in Patagonia [13]; Francisco Hernández (1570–1577) [14], Nicolas Monardes (1574) [15] and Martín Sessé (1788–1796) [16, 17] in Mexico; Paul Hermann in Surinam and the Guianas region [18]; Hans Sloane in Jamaica (1687–1689) [19]; Hipólito Ruiz and Antonio Pavón (1777–1786) and Joseph de Jussieu (1735–1771) in Peru [20, 21]; José Celestino Mutis (1782–1808) in Colombia [22]; Charles Plumier (1689–1697) in Martinique and Haiti [23]; Jean-Baptiste C. F. Aublet (1762) in the French Guiana [24], and Charles-Marie de la Condamine (1735–1744) from the Peruvian Andes to the Atlantic Ocean through the Amazon river [25].

Among all these names, the German Alexander von Humboldt was undoubtedly the most prominent naturalist of his time. Alongside the French Botanist Aimee Bonpland, Humboldt conducted an expedition to the Spanish American Colonies between 1799 and 1804, collecting more than 6000 plant specimens. Humboldt was a pioneer, inspiring an entire generation of naturalists who succeeded him to explore South American biodiversity. The information gathered by Humboldt greatly contributed to the growing knowledge of South American biodiversity, led to significant advances in understanding the American continent’s natural history and, most importantly, radically changed the view about the importance of nature for the life of the planet [26].

Although practically only male names are cited among naturalists, female naturalists had significant participation in constructing scientific knowledge regarding plant biodiversity for centuries. Women’s trajectories are often neglected by the history of science, and the scarcity of female names constructs the image that sciences were exclusively a male practice [27]. The most prominent female naturalist is certainly the German Maria Sibylla Merian, who published a book on insects and plants during an expedition in Surinam between 1699 and 1701 [28]. Nonetheless, Mariath and Baratto [27] found 28 female naturalists who participated in scientific expeditions from the seventeenth to the nineteenth century, recording or illustrating useful plants.

### The Baron de Santa-Anna Nery: a propagandist of the Brazilian Amazon

Besides the naturalists, propagandists played an important role in promoting Brazilian natural resources. Often, governments would hire intellectuals to design and promote the region's characteristics abroad, aiming to promote the modernization of Brazil [29].

**Fig. 1:**
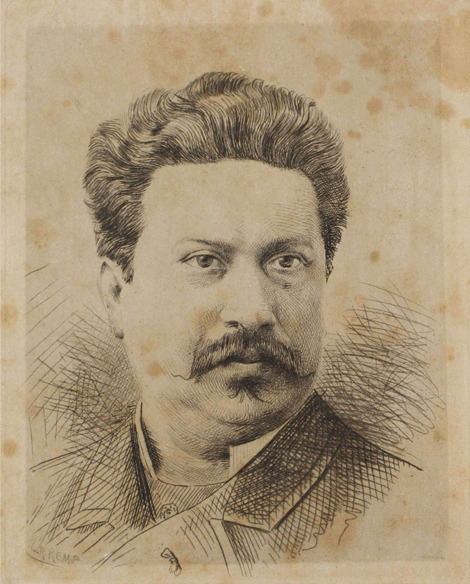
Portrait of Baron de Santa-Anna Nery (1848–1901) [32]

One of them was Frederico José de Santa-Anna Nery (1848–1901) (Fig. 1), who was born in the Brazilian Amazon, in Belém, the capital of the state of Pará, Brazil, but at the age of 14, he moved to Paris, France, and lived in Europe until his death [30]. He earned a Bachelor's degree in Letters and a Doctorate in Law from the University of Rome, and he worked for French newspapers and magazines (“L’Événement Écho de Paris”, “L’Opinion”, “Le Fígaro”, “L’América", “Republique Française”), as well as Italian (“La Tribuna”, “Libertá”, “Journal de Rome”, “Il Século”) and even Brazilian publications (“Jornal do Commercio”). He was the owner and editor of the “Revue du Monde Latin” and the director of the magazine “Le Brésil”, publications that always sought to portray a positive image of Brazil and Latin American countries [30, 31].

Baron de Santa-Anna Nery referred to himself as a "volunteer propagandist" for Brazil in Europe, serving as an immigration agent to publicize the living conditions in the Amazonas province, advocating for the development and modernization of the Amazon [29, 33]. Due to his family's elite status in Manaus and Belém, he had the advantage of establishing political relationships with influential figures in the Brazilian government at the time, which sought to promote the colonization of the Amazon through European immigration [29]. An example of his political influence was when Santa-Anna Nery defended Brazil's image and interests in the territorial dispute over part of the Amazon (currently the state of Amapá, Brazil) with France (French Guiana). The French naturalist Henri Coudreau, who initially advocated for his country's interests, upon meeting and becoming friends with Santa-Anna Nery, became an ally of Brazil. In fact, Coudreau was even hired by the state of Pará to map the course of rivers that were still unknown, and this cartographic data was crucial for Brazil's defense of the contested Amazonian territory. Santa-Anna Nery played a significant role in this Brazilian victory in 1900, after the international arbitration by Switzerland, mainly due to his ability to discover ancient documents validating Brazil's rights over the territory, as well as his extensive sociability and influence within the political and social circles of Europe and Latin America [30, 34].

**Fig. 2:**
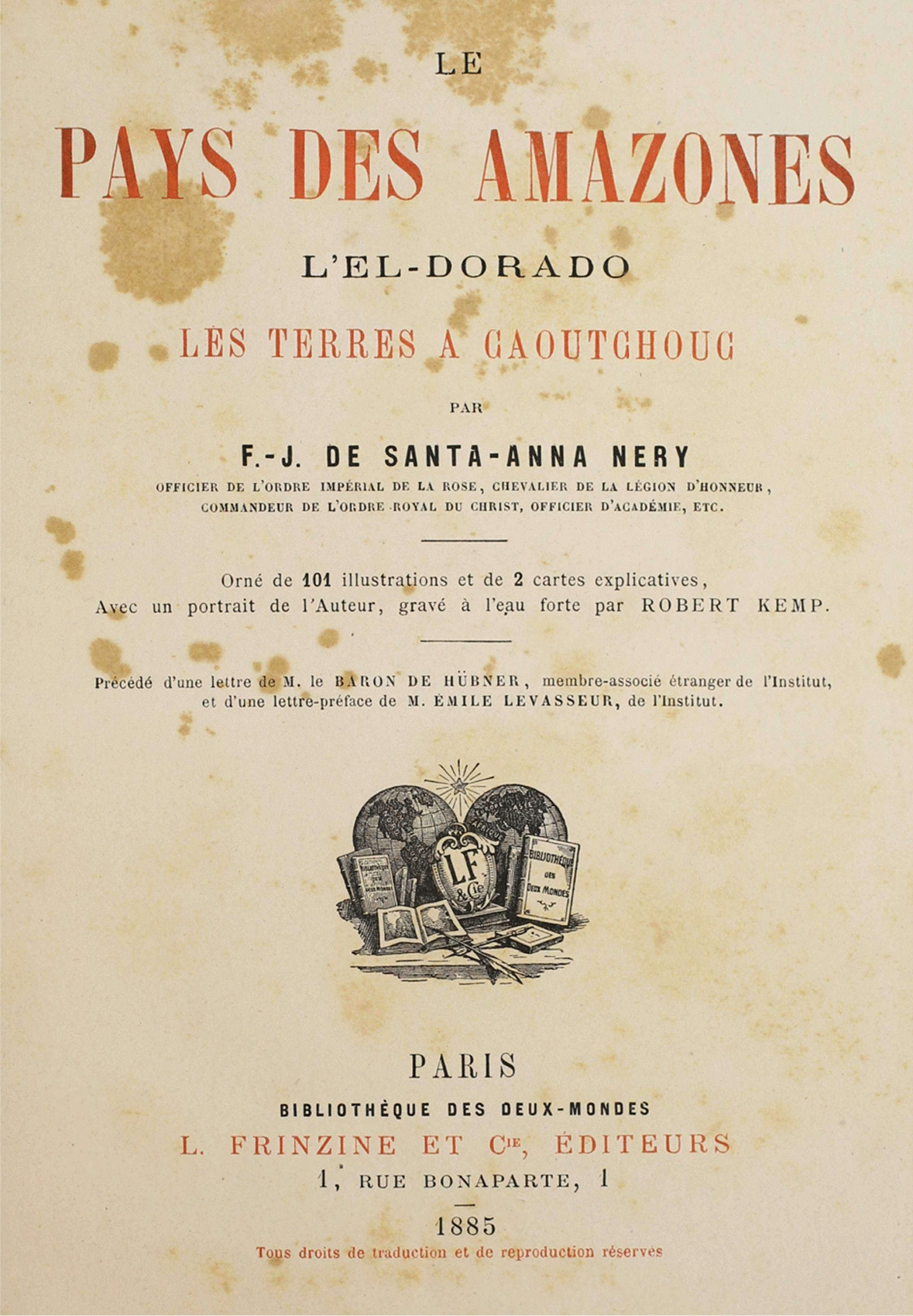
Cover page from the Baron de Santa-Anna Nery’s “Le Pays des Amazones,” first published in 1885 [32]

Santa-Anna Nery’s most famous book is "Le Pays des Amazones" (The Lands of the Amazons) [32], first published in 1885 (Fig. 2), which was sponsored by the government of Manaus with the aim of promoting the state of Amazonas to the outside world. Interestingly, this work was written in French and was only translated into Portuguese in 1979 by Ana Manzur Spira. In this book, the author portrays all the Amazonian regionalism and the work is divided into three focuses: The first is to highlight the natural abundance of the Amazon as a possibility to acquire wealth; the second is to dispel negative ideas about the region regarding climate and tropical diseases; and the third is to showcase the modernized urban spaces and economic opportunities that could be found in the region [33].

In "Le Pays des Amazones", Santa-Anna Nery depicted the fauna, flora, minerals, habits, and lifestyles of the riverside dwellers, indigenous people, and immigrants. He extensively explores the rubber economy and the possibilities of economic progress based on agriculture and foreign labor, drawing from his own experiences and historical documents from prominent travelers who ventured through the Amazon, including figures such as Manuel Rodríguez (1684), Charles-Marie de la Condamine (1735–1744), Alexander von Humboldt (1799–1804), Spix and Martius (1817–1820), Louis Agassiz (1865–1866), among others [35]. In the book, the author dedicated a chapter to introduce and report on the plant kingdom and its relationship with humans, from the economic value of woods to plants used for treating diseases. The author systematizes the classification of plants according to their function, briefly discussing each category: construction woods used in common, naval, or civil construction; woods for carpentry and furniture manufacturing; edible plants; spices and aromatic plants; textile fibers; plant-based dyeing, tanning, and toning materials; oilseeds; medicinal substances; gums, gum-resins, resins, oil-resins, balms, and essences; vegetable ivory and concentrated juices [33, 35].

The aim of this work is to understand the historical context and ethnobotanical value of the plant species from the Brazilian Amazon at the end of the nineteenth century through an analysis of the book "Le Pays des Amazones" (1885) by Baron de Santa-Anna Nery. Furthermore, we aim to rescue the historical importance of the Baron de Santa-Anna Nery, a very influential Brazilian propagandist abroad at the end of the nineteenth century, but that was forgotten nowadays. This is the first analysis involving the comparison of historical ethnobotanical information with contemporary scientific evidence regarding the plants described in his book.

## Methods

The original book “Le Pays des Amazones” published in 1885 [32] was consulted in the online catalog of the Biblioteca Brasiliana Guita e José Mindlin—PRCEU/USP (https://www.bbm.usp.br). The original 3rd edition [36] and its translated version into Portuguese [35] were consulted to compare information. After reading the book, all information about plants classified as medicinal or those that had therapeutic properties, or those with some other uses, was systematized in a table organized by botanical description, page number, vernacular name, plant part, origin, traditional uses and observations. The updated botanical names and the origin of each plant were verified in Plants of The World Online (https://powo.science.kew.org/), Tropicos (https://tropicos.org/home) or Flora e Funga do Brasil (http://floradobrasil.jbrj.gov.br/). Origin classification of the plants (Brazilian native, exotic, naturalized, or cultivated) followed the terminology adopted by Moro et al. [37]. Traditional uses were classified as following: (a) medicinal, (b) edible plants (food and beverages), (c) constructions (civil, naval and railway) and buildings, (d) spices and condiments, (e) ornaments and objects (ropes, brooms, rags, hammocks, nets, violin strings, ship wedges), (f) carpentry and furniture, (g) textile fibers (ropes, rags, linen, fabric, lace), (h) resins, oleoresins, gums, oils, balms, latex, rubber, essences for perfumery, (i) pigments, dyeing and tanning, (j) hunting and fishing, (k) lighting, (l) other uses (latex smoking, "tucupi" extraction, rituals, animal fodder, press, "plant milk"). Finally, using the scientific name of medicinal plants alone or in combination with their popular use as keywords, a search was carried out in Pubmed, ScienceDirect and Google Scholar in order to indicate current pharmacological studies that provide evidence about the described traditional uses, as well as general biological activities that have been studied.

## Results and discussion

In the book "Le Pays des Amazones" [32, 35, 36], a total of 156 plant species were identified. Out of these, 132 species had their scientific names updated, while the remaining 24 lacked sufficient information. These species belong to 45 different families, with Fabaceae (23) and Arecaceae (17) the most represented, followed by Euphorbiaceae (10), Lauraceae (7) and Malvaceae (6); the remaining families each contained between 1 to 5 species.

Regarding the origin of the plants, 109 species were Brazilian native, 10 were exotic, 7 were cultivated and 6 were naturalized. It is important to clarify the terminology regarding the origin: (a) Native species are those naturally occurring in a specific location, with their presence in the area attributed to their own dispersal capacity and ecological competence, without human influence; (b) exotic or alien species are those that would not naturally occur in a given geographic region without human transport (intentionally or accidentally) to the new area; (c) cultivated plants are exotic species that may eventually reproduce in the environment where they were introduced. However, they are not capable of sustaining an autonomous population in the long term, requiring human management (cultivation); (d) naturalized plants are exotic species that can consistently reproduce in the location where they were introduced, establishing a self-perpetuating population without the need for direct human intervention. Nevertheless, they have not dispersed far from the introduction site and remain restricted to that location [37].

Considering only the medicinal plants, 36 species were identified (Table 1), among them 24 had their therapeutic indications defined by Baron de Santa-Anna Nery, while the other 12 were only assigned as “medicinal” not specified for what therapeutic purposes they were used, classified by us as “undefined.” The main medicinal indications reported were astringent (5), purgative/laxative (5), stimulant and tonic (5), vermifuge (4), febrifuge (3), sudorific (3), emetic, diuretic and antidysenteric (2). The traditional uses reported by Santa-Anna Nery are supported by pharmacological studies in approximately 75% (18) of the medicinal species in relation to those with defined purposes.

**Table 1:** Medicinal plants described in the book "Le Pays des Amazones" [The Lands of the Amazons] (1885) by Frederico José de Santa-Anna Nery (1848–1901)

Botanical family and species^a^	Brazilian vernacular name	Page book	Part	Origin	Traditional use	Pharmacological evidence for species or genus
Annonaceae						
* Xylopia* sp.	embira	100, 105	Fiber, seed	Native	Purgative	Cytotoxic [38, 39]; antimicrobial [40]
Asteraceae						
* Acmella oleracea* (L.) R.K. Jansen (= *Spilanthes oleracea* L.)	agrião-do-pará	104	Not mentioned	Naturalized	Anti-scorbutic, analgesic (for toothaches), antipyretic (for intermittent fever)	Anti-scorbutic^†^ [41]; oral analgesic^†^ [42]; anti-inflammatory^†^ [43]; gastroprotective [44]; diuretic [45]
Bignoniaceae						
* Jacaranda copaia *(Aubl.) D.Don (= *Bignonia copaia* Aubl.)	not mentioned	104	Fruit, stem bark	Native	Emetic, purgative; fruit is antisyphilitic	Antileishmanial [46]; antimalarial [47]
* Tabebuia insignis* (Miq.) Sandwith (= *Tecoma insignis* Miq.)	ipê-tabaco	106	Stem bark	Native	Purgative; dust causes sneezing	Antihyperuricemic, anti-inflammatory [48, 49]; antinociceptive [50]
* Tecoma* sp.	ipê branco	106	Stem bark	Naturalized	Purgative	Antidiarrheal [51]
Caricaceae						
* Carica papaya* L.	mamoeiro	102	Unripe fruit	Naturalized	Vermifuge	Anthelmintic^†^ [52, 53]; hypoglycemic [54]; immunomodulatory [55]; antithrombocytopenic [56]
Chrysobalanaceae						
* Couepia* sp. (= *Pleraginea* sp.)	pajurá	106	Seed	Native	astringent	Cytotoxicity, antioxidant, antibacterial [57, 58]
Dipterocarpaceae						
* Vateria* sp. [= Vateria guyanensis*]	pau-de-espinhas	104	Seed	Exotic	Acne treatment with vinegar	Wound healing [59]; antiepileptic [60]; antioxidant, antidiabetic, cytotoxic [61]
Erythroxylaceae						
* Erythroxylum coca* Lam.	ipadu, coca	91	Leaf	Native	Indigenous people chewed to deceive hunger; stimulant and tonic	Stimulant^†^ [62, 63]
Fabaceae						
* Bowdichia virgilioides* Kunth	sucupiraçu, sucupira pérola	106	Wood, stem bark	Native	Astringent	Wound healing^†^ [64]; gastric protective agent^†^ [65]; antihyperglycemic [66]; antinociceptive [67, 68]; anti-inflammatory [68]
* Cassia grandis* L.f. (= *Cassia brasiliana* Lam.)	jeneúna	104	Not mentioned	Native	Medicinal (undefined)	Hypoglycemic [69]; antioxidant [70]
*Copaifera guianensis* Desf.	copaíba oil, copahu	105	Oil	Native	Medicinal (undefined)	Anti-inflammatory [71]; antimicrobial [72]; anticariogenic, antiparasitic [73]
* Copaifera langsdorffii* var. *langsdorffii* Desf. (= *Copaifera nitida* Mart. ex Hayne)	copaíba oil	183	Oil	Native	Medicinal (undefined)	Anti-inflammatory, analgesic, wound healing [71, 74–77]
* Dipteryx odorata* (Aubl.) Forsyth f.	cumaru, favas-tonca	98,105,180	Seed, oil, coumarin (isolated compound)	Native	Medicinal oil (undefined)	Anti-inflammatory, antiplatelet aggregation [78]
* Tamarindus indica* L.	tamarindo	105	Fruit pulp	Cultivated	Laxative	Laxative^†^ [80, 81]; wound healing [82]; antimicrobial, anti-inflammatory, antioxidant [83]
Krameriaceae						
* Krameria argentea* Mart. ex Spreng.	ratânia	104	Not mentioned	Native	Astringent	Astringent^†^; intestinal diseases^†^ [84–86]; cytotoxic [87]; antinociceptive, anti-arthritic [88]
Lauraceae						
* Aniba puchury-minor* (Mart.) Mez (= *Nectandra puchury-major* (Mart.) Nees & Mart. = *Nectandra puchury-minor* (Mart.) Nees & Mart.)**	puxuri	180	Not mentioned	Native	Medicinal (undefined)	Antifungal, anti-inflammatory [89]
* Ocotea sassafras *(Meisn.) Mez (= *Mespilodaphne sassafras* Meisn.)	canela-sassafrás, sassafrás essence	105, 108	Root	Native^c^	Aromatic root used in therapy (undefined) and essences	Antifungal [90]; anticoagulant [91]
Lecythidaceae						
* Lecythis ollaria* L.	sapucaia, camari-macaco	85,103	Almonds	Exotic	Food and medicinal (undefined)	Wound healing [92]; Selenium poisoning (e.g., alopecia) [93, 94]
Loganiaceae						
* Spigelia anthelmia* L.	not mentioned	104	Not mentioned	Native	Poisonous when fresh and vermifuge when dry	Anthelmintic^†^ [95, 96]; cardiac contraction [97, 98]; neuromuscular blockade [99]
Malvaceae						
* Theobroma cacao* L.	cacaueiro	103	Cacao butter	Native	Medicinal (undefined)	Antioxidant [100, 101]; anti-inflammatory [102]; neuroprotective [103]; cardioprotective, hepatoprotective and nephroprotective combined with doxorubicin [104]; antialopecia [105]
Meliaceae						
* Carapa procera* DC. (= *Carapa guyanensis* Oliv.)	andiroba, carapa oil	104, 105	Stem Bark	Cultivated	Bitter Tonic, febrifuge	Antimicrobial^†^ [106]; antimalarial^†^ [107, 108]
* Cedrela odorata* L. (= *Cedrela guianensis* A.Juss.)	cedro-branco, acaju-amargo, acaju-fêmea	84, 105	Stem bark	Native	Bitter tonic, febrifuge	Antimalarial^†^ [109, 110]; antileishmanial^†^ [111]; insecticidal [112]; antidiabetic [113]; anti-inflammatory, anti-allergic [114]
Moraceae						
* Ficus gomelleira* Kunth & C.D.Bouché (= *Ficus doliaria* (Miq.) Mart.)	gameleira	106	Sap	Native	Vermifuge	Antiparasitic^†^ [115, 116]
Myristicaceae						
* Virola bicuhyba* (Schott) Warb. (= *Myristica bicuhyba* Schott.)	bicuíba	103	Oil	Native	Medicinal (undefined)	Gastroprotective [117]; antimicrobial [118]; wound healing [119]; antinociceptive, anti-inflammatory [120]
* Virola* sp. (= *Myristica* sp.)**	sucuuba	106	Resin	Native	Vermifuge	Antiprotozoal^†^ [121–123], gastroprotective, cytotoxic, antiprotozoal, antimicrobial [124]
Myrtaceae						
* Eugenia cerasiflora* Miq. (= *Eugenia lucida* Lam)	murta	106	Stem bark	Native^d^	Astringent	Astringent^†^ [125]; healing of gastric ulcers^†^ [126]; wound healing^†^ [127]
* Psidium guajava* L. (= *Psidium pomiferum* var. *sapidissimum* (Jacq.) DC.)	goiabeira	104	Root, leaf	Naturalized	Astringent, antidysenteric	Antidiarrheal^†^ [128]; antimicrobial, antiprotozoal [129]; cytotoxic [130]
Rubiaceae						
* Carapichea ipecacuanha* (Brot.) L. Andersson (= *Cephaelis ipecacuanha* (Brot.) Willd.)	ipecacuanha, poaia	104	Not mentioned	Native	Emetic, expectorant, diaphoretic (sudorific)	Expectorant^†^ [131], emetic^†^ [132, 133], immunomodulatory [134]
* Genipa americana* L. (= *Genipa brasiliensis* (Sprend.) Baill)	jenipapo	106	Fruit	Native	Medicinal (undefined)	Antiplatelet, anti-inflammatory [135]; neuroprotective [136]; antioxidant, anti-glycant [137]; antithrombotic [138]; anticonvulsant [139]; trypanocidal [140]
Sapindaceae						
* Paullinia cupana* Kunth. (= *Paullinia sorbilis* Mart.)	guaraná	92	Seed	Native	Stimulant, enhancement of intellectual functions, antidysenteric	Central Nervous System (CNS) stimulant^†^ [141–143], anti-adipogenic [144]; anti-obesity [145, 146]; anti-inflammatory [147]
Sapotaceae						
* Pradosia lactescens* (Vell.) Radlk) (= *Chrysophyllum glycyphloeum* Casar)	buranhém	105	Stem bark	Native	Medicinal (undefined)	Anti-wrinkling, anti-melanogenic [148]
Smilacaceae						
* Smilax glauca* Walter (= *Smilax sarsaparilla* L.)**	salsaparrilha	104	Root	Exotic	Diuretic, sudorific	Diuretic^†^ [149, 150]; antihyperlipidemic [151]; hypoglycemic [152]; anti-inflammatory, analgesic [153]
* Smilax schomburgkiana* Kunth or *S. longifolia* Rich. (= *S. syphilitica* Griseb. or *S. syphilitica* Mart., respectively)^b^	salsaparrilha-do-brasil	182	Root	Native	Diuretic, sudorific	Diuretic^†^ [149, 150]; antihyperlipidemic [151]; hypoglycemic [152]; anti-inflammatory, analgesic [153]
Solanaceae						
* Brunfelsia uniflora* (Pohl) D.Don (= *Brunfelsia hopeana* (Hook.) Benth)	jeratacaca	110^e^	Not mentioned	Native	Against snake bite	Anti-venom^†^ [154], anti-inflammatory [155]; larvicidal [156]; antimicrobial [157]
Zingiberaceae						
* Zingiber officinale* Roscoe (= *Amomum zinziba* Hill)	gengibre	98	Rhizome	Cultivated	Spice, therapeutic (undefined), edible (ginger beer)	Nephroprotective [158]; anti-inflammatory [159]; cytotoxic [160]; antioxidant, antihyperalgesia [161]; antiasthmatic [162]

*Unrecognized name/spelling cited exactly in the book

**According to the 3rd edition (1899)

^†^Pharmacological correlation with traditional use

^a^updated botanical names (in parenthesis = terminology as originally described by Santa-Anna Nery)

^b^Originally cited as *Smilax syphilitica*, without authorship definition

^c^Native from Southeast Brazil

^d^Native from the sea coast Brazil

^e^Only included in the 3rd edition (1899)

A total of 106 useful plants (non-medicinal) were included in Table 2, 9 with undefined uses and 97 with some traditional use, like edible species (27), constructions and buildings (15), spices and condiments (12), ornaments and objects (20), carpentry and furniture (10), textile fibers (11), resins, oleoresins, gums, oils, balms, latex, rubber and essences for perfumery (39), pigments, dyeing and tanning (9), hunting and fishing (5), lighting (3), among other uses.

**Table 2:** Useful plants (except medicinal plants) described in the book "Le Pays des Amazones" [The Lands of the Amazons] (1885) by Frederico José de Santa-Anna Nery (1848–1901)

Botanical family and species^a^	Brazilian vernacular name	Page book	Part	Origin	Traditional use
Anacardiaceae					
* Anacardium occidentale* L.	caju, cajueiro	98,104, 106,223	Fruit and nut, resin	Native	Food, preparation of a beverage similar to wine
* Spondias dulcis* Parkinson	cajazeiro, cajá, pomo-de-citera	87	Fruit, wood	Exotic	Carpentry
Annonaceae					
* Annona* sp. (= *Rollinia* sp.)	beribá	107	Sap	Native	Balm
* Xylopia* sp.	embira	100	Fiber, seed	Native	Textile, objects (ropes and brooms)
Apocynaceae					
* Aspidosperma* sp.	pau-cetim	88	Wood	Native	Carpentry
* Couma utilis* (Mart.) Müll.Arg. (= *Collophora utilis* Mart*.)*	sorva, sorveira	98, 107, 188	Fruit, sap	Native	Food; resin used as varnish by indigenous people
* Hancornia speciosa* Gomes	mangaba	188, 192	Fruit	Native	Food
* Macoubea guianensis* Aubl.	macacú	100	Unripe fruit	Native	Red blood dye that darkens when exposed to urine vapor
Arecaceae					
* Astrocaryum vulgare* Mart.	tucum	102,179	Fruit, fiber, wood,	Native	Fruit oil for lighting and industrial uses; fiber for utensils and objects (ropes and nets), wood for construction
* Astrocaryum murumuru* Mart.	murumuru	102	Fruit, oil	Native	Food
* Astrocaryum jauari* Mart.	jauari	102	Fruit, oil	Native	Food
* Astrocaryum tucuma* Mart.	tucum, tucumá	67,99, 166	Fruit, fiber	Native	Ropes, hammocks and fishing nets, ornaments
* Attalea funifera* Mart.	piaçaba, piaçava	100, 180	Bark fiber, fruit	Native	Ropes, brooms, rags, oil
* Attalea phalerata* Mart. ex Spreng. (= *Attalea excelsa* Mart.)	urucuri, iuauaçú	190	Fruit	Native	Latex smoking
* Bactris gasipaes* var. *gasipaes* Kunth (= *Guilielma speciosa* Mart.)	pupunha	97	Fruit, leaf	Native	Food, fibers for textile material
* Desmoncus* sp. (= *Desmonchus* sp.)	jacitara	156	Stem	Native	Stems are employed in the crafting of an elastic tube known as "tipiti," which is utilized for the extraction of "tucupi," a liquid derived from cassava root
* Elaeis oleifera* (Kunth) Cortés (= *Elaeis melanococca* Mart.)	palmeira ciaué	102	Seed (almond)	Native	Food, oil similar to palm oil
* Euterpe oleracea* Mart.	coco de juçara	102	Fruit, oil	Native	Food
* Leopoldinia piassaba* Wallace	not mentioned	180	Fiber, fruit	Native	Ropes, brooms, rags, oil
* Leopoldinia pulchra* Mart.	jaraúba	101	Not mentioned	Native	Yellow dye
* Manicaria saccifera* Gaertn.	urucuri, iuauaçú	190	Fruit	Native	Latex smoking
* Mauritia flexuosa* L.f.	miriti	100, 102	Fiber, fruit	Native	Textile fiber, oil in spices
* Oenocarpus bacaba* Mart.	bacaca	102	Fruit, oil	Native	Food, fruit in indigenous beverages, and oil as a spice
* Onenocarpus bataua* Mart.	patuá	102	Fruit, oil	Native	Food, fruit in indigenous beverages, and oil as a spice
* Phytelephas macrocarpa* Ruiz & Pav. (= *Elephantusia macrocarpa* (Ruiz & Pav.) Willd.	marfim vegetal (plant ivory)	108	Seed	Native	Objects and ornaments
Bignoniaceae					
* Handroanthus chrysanthus* (Jacq.) S.O.Grose (= *Tecoma chrysantha* (Jacq.) DC.)	ipê, pau-d'arco	84	Wood	Exotic	General construction
Bixaceae					
* Bixa orellana* L.	urucu	100	Fruit	Native	Red dye
Boraginaceae					
* Cordia* sp.	louro negro, comum, amarelo, cheiroso, branco, vermelho	85	Wood	Native	General construction
Bromeliaceae					
* Ananas comosus* var. *comosus* (L.) Merr. (= *Bromelia ananas* L.)	ananás, abacaxi	98, 100, 165,	Fruit, fiber	Native	Food, textile
* Ananas comosus* var. *bracteatus* (Lindl.) Coppens & F.Leal (= *Bromelia sagenaria* Arruda)	curauá	100	Not mentioned	Native^b^	Linen, fabric, lace, violin strings
Burseraceae					
* Protium glabrum *(Rose) Engl. (= *Icica glabra* Rose)	pau-de-breu	107	Resin	Exotic	Not mentioned
* Protium icicariba* (DC.) Marchand (= *Icica icicariba* DC.)	icicariba, resin elemi (french)	107	Oleoresin	Native^c^	Not mentioned
Calophyllaceae					
* Calophyllum brasiliense* Cambess.	guanandi, lantim, jacaré-uba	102, 107	Oil, balm	Native	Spice
Campanulaceae					
* Siphocampylus* sp.**	not mentioned	206	Not mentioned	Native^d^	Latex for rubber
Caryocaraceae					
* Caryocar brasiliense* A.St.-Hil. (= *Caryocar brasiliensis*)	piquiá, piqui	84, 102	Fruit, wood	Native	Food or spice, construction
Clusiaceae					
* Symphonia globulifera* L.f.	unani	107	Resin	Native	Not mentioned
* Platonia insignis* Mart.	parcouri, bacuri	83	Wood, gum	Native	Civil and naval construction
Convolvulaceae					
* Ipomoea batatas* (L.) Lam. (= *Convolvulus batatas* L.; *Batatas edulis* (Thunb.) Choisy.)	batata-doce	93, 94, 97	Tuber	Naturalized	Food
Dioscoreaceae					
* Dioscorea* sp.	inhame, cará	94, 97	Tuber, leaf	Native	Tuber used as food and leaves for animal fodder
Euphorbiaceae					
* Hevea guianensis* Aubl. (= *Siphonia elastica* Forsyth f.)	seringueira, rubber tree	102, 188 189, 191	Latex, oil, gum (juice)	Native	Objects in general, oil used in soap production and press
* Hevea benthamiana* Müll.Arg. (= *H. discolor* Spruce ex Pax)**	seringueira, rubber tree	203	Gum (juice)	Native	Objects in general
* Hevea brasiliensis* (Willd. ex A.Juss.) Müll.Arg.**	seringueira, rubber tree	203	Gum (juice)	Native	Objects in general
* Hevea guianensis* var. *lutea* (Spruce ex Benth.) Ducke & R.E.Schult. (= *H. lutea* (Spruce ex Benth.) Müll.Arg.; *H. apiculata* Spruce ex Baill.)**	seringueira, rubber tree	203	Gum (juice)	Native	Objects in general
* Hevea pauciflora* (Spruce ex Benth.) Müll.Arg.**	seringueira, rubber tree	203	Gum (juice)	Native	Objects in general
* Hevea pauciflora* var. *pauciflora* (= *H. membranacea* Müll.Arg.)**	seringueira, rubber tree	203	Gum (juice)	Native	Objects in general
* Hevea rigidifolia* (Spruce ex Benth.) Müll.Arg.**	seringueira, rubber tree	203	Gum (juice)	Native	Objects in general
* Hevea spruceana* (Benth.) Müll.Arg.**	seringueira, rubber tree	203	Gum (juice)	Native	Objects in general
* Manihot carthagenensis* subsp*. glaziovii* (Müll.Arg.) Allem*.* (= *Manihot glaziovii* (Müll.Arg.) Allem.)**	maniçoba	203	Not mentioned	Native	Rubber
* Manihot esculenta* Crantz. (= *Manihot aypi* Spruce.; *Manihot utilissima* Pohl.)	mandioca-doce, macaxeira, mandioca-amarga, mbai-ybai	93,94,95,96	Root	Native	A mixture of wheat flour with cassava flour results a bread inferior to bread made solely with wheat flour, alcohol production, tapioca, mussacha, and tucupi
Fabaceae					
* Abarema cochliacarpos* (Gomes) Barneby & J.W.Grimes (= *Pithecellobium auaremotemo* Mart.)	barbatimão	101	Bark	Native^e^	Astringent used in dyeing
* Anadenanthera peregrina* (L.) Speg. (= *Mimosa acacioides* Benth.)**	paricá	277	Not mentioned	Native	Like tobacco in puberty ritual
* Andira* sp.	andira-uixi	86	Wood	Native	Carpentry and fine furniture
* Bowdichia virgilioides* Kunth	sucupiraçu, sucupira pérola	83	Wood, Bark	Native	Ship keels
* Centrolobium* sp.	muiraguatiara	87	Not mentioned	Native	Carpentry and fine furniture
* Centrolobium paraense* Tul.**	muirapinina, pau-tartaruga, pau-letras	87	Not mentioned	Native	Carpentry and fine furniture
* Dalbergia nigra* (Vell.) Allemão ex Benth.	jacarandá-cabiúna	87	Wood	Native	Furniture and objects
* Dipteryx odorata* (Aubl.) Forsyth f.	cumaru, favas-tonca	98, 105,180	Seed, coumarin, oil	Native	Perfumery; spices, seeds used to perfume tobacco
* Geoffroea spinosa* Jacq. (= *Geoffroya superba* Humb. & Bonpl.)	umari	88	Wood	Native	Not mentioned
* Haematoxylum campechianum* L.	pau-campeche	101	Not mentioned	Cultivated	Dye
* Hymenaea courbaril* L.	jatobá	107	Resin	Native	Indigenous people produce ornaments
* Inga affinis* DC. or *Pithecellobium dulce* (Roxb.) Benth. (= *Inga dulcis*)	ingá	98	Fruit	Native	Food
* Inga* sp.	ingarana	87	Wood	Native	Carpentry and fine furniture
* Paubrasilia echinata* (Lam.) Gagnon, H.C.Lima & G.P.Lewis (= *Caesalpinia echinata* Lam.)	pau-brasil	101	Not mentioned	Native	Dye
* Peltogyne* sp. (= Peltogyne macrocarpus; P. macrolobium**)*	guarabu, pau-roxo	85	Wood	Native	General construction
* Peltogyne venosa* (Vahl) Benth.	pau-roxo do Amazonas	88	Wood	Native	Carpentry and fine furniture
* Pentaclethra macroloba* (Willd.) Kuntze. (= *Pentaclethra filamentosa* Benth.)	pau-mulato	88	Wood	Native	Carpentry and fine furniture
* Swartzia panacoco* var.* panacoco* (Aubl.) R.S.Cowan (= *Swartzia tomentosa* (Willd.) DC.)	pau-ferro	84	Wood	Native	General construction
* Vouacapoua americana* Aubl. (= *Andira aubletii* Benth.)	acapú	86	Wood	Native	General construction
Humiriaceae					
* Humiria balsamifera* var. *floribunda* (Mart.) Cuatrec. (= *Humiria floribunda* Mart.)	umiri, nieri das colônias	86, 102, 108	Wood, fruit, oil	Native	General construction, food, substitute for peru balsam
Hypericaceae					
* Vismia guianensis* (Aubl.) Pers.	pau-de-lacre, pau-de-sangue	106	Resin	Native	Juices with a strong odor and bitter taste
Lauraceae					
* Cinnamomum* sp.	canela	98	Not mentioned	Exotic	Spice
* Dicypellium* sp*.* Nees & Mart.	pau-rosa	88	Wood	Native	Not mentioned
* Licaria guianensis* Aubl.	cravo	98	Not mentioned	Native	Spice
* Mespilodaphne quixos* (Lam.) Rohwer (= *Mespilodaphne pretiosa*)	pau-precioso	88	Wood, bark, seed	Exotic	Pharmacy and perfumery
* Mezilaurus ita-uba* (Meisn.) Taub. ex Mez (= *Acrodiclidium ita-uba* Meisn.)	itaúba, pau pedra	85	Wood	Native	Not mentioned
Lecythidaceae					
* Bertholletia excelsa* Bonpl.	castanha-do-pará, castanheira comum, castanheiro do brasil	24, 103, 183, 184	Fruit, wood, nut, oil	Native	Common and naval construction; hard shell for tow; nut and oil for food
Loganiaceae					
* Strychnos toxifera* R.H.Schomb. ex Lindl.	urari	104	Not mentioned	Native	Indigenous people use it to prepare curare and apply it to arrows for fishing
Malvaceae					
* Sterculia pruriens* (Aubl.) K.Schum. (= *Sterculia ivira* Sw.)	tururi	100	Not mentioned	Native	Fibers for export, ropes
* Theobroma bicolor* Bonpl.	cacaueiro	90	Not mentioned	Native	Food
* Theobroma cacao* L.	cacaueiro	103	Seed butter	Native	Perfumery
* Theobroma speciosum* Willd. ex Spreng.	cacaueiro	90	Not mentioned	Native	Food
* Theobroma sylvestre* (Aubl. ex Mart.)	cacaueiro	90	Not mentioned	Native	Food
* Urena lobata* L.	uaicina	100	Not mentioned	Native	Fibers for export, ropes
Marantaceae					
* Ischnosiphon arouma* (Aubl.) Körn.(= *Maranta arouma* Aubl.)	guarumá	156	Stem	Native	Stems are employed in the crafting of an elastic tube known as "tipiti," which is utilized for the extraction of "tucupi," a liquid derived from cassava root
* Maranta arundinacea* L.	araruta	93, 97	Root	Cultivated	Food
Meliaceae					
* Carapa procera* DC. (= *Carapa guyanensis* Oliv.)	andiroba, óleo de carapa	104, 105	Oil	Cultivated	Lighting, soap production
* Cedrela* sp.	cedro-batata	85	Bark, wood	Native	Construction
* Cedrela odorata* L. (= *Cedrela guianensis* A.Juss.)	cedro-branco, acaju-amargo, acaju-fêmea	84, 105	Wood	Native	Civil and naval construction
Moraceae					
* Artocarpus altilis* (Parkinson) Fosberg (= *Artocarpus incisus* (Thunb.) L.f.)	fruta-pão	93	Fruit	Naturalized	Not mentioned
* Maclura tinctoria* (L.) D.Don ex G.Don	tatajuba-de-tinta	101	Not mentioned	Native	Yellow dye
Musaceae					
* Musa x paradisiaca* L. (= *Musa x sapientum* L.)	bananeira, banana	93	Fruit	Cultivated	Food
Myristicaceae					
* Virola bicuhyba* (Schott) Warb. (= *Myristica bicuhyba* Schott.)	bicuíba	102, 103	Oil	Native	Lighting
Orchidaceae					
* Vanilla planifolia* Andrews (= *Vanilla sativa* Schiede.)	baunilha	98	Not mentioned	Native	Spice
Rhizophoraceae					
* Rhizophora mangle* L.	mangue vermelho	101	Bark	Native	Tanning
Rubiaceae					
* Genipa americana* L. (= *Genipa brasiliensis* (Sprend.) Baill)	jenipapo	87	Wood, fruit	Native	Furniture, food
Rutaceae					
* Galipea* sp.	guariúba	86	Wood	Native	Not mentioned
Sapindaceae					
* Magonia* sp*.* (= *Phaeocarpus* sp.)	tingui	171	Bark, leaf, fruit	Native	Narcotic in fishing
* Paullinia cupana* Kunth (= *Paullinia sorbilis* Mart.)	guaraná	92, 180	Seed (grains)	Native	Food
* Paullinia pinnata* L. (= *Paullinia grandiflora* Cambess.)	cruapé-vermelho, turari	171	Not mentioned	Native	Narcotic in fishing
* Serjania ferruginea* (Lindl.) Mabb. (= *Serjania cuspidata* Cambess.)	timbó	171	Oil, juice	Native^f^	Narcotic in fishing, narcotic
Sapotaceae					
* Manilkara bidentata* (A.DC.) A. Chev. subsp. *bidentata* (*Mimusops balata* (Aubl.) C.F.Gaertn.)	maçaranduba, muirapiranga,	84,88, 192,101	Wood, sap, bark	Native	Wood for railroad ties, ship wedges; gutta-percha as a substitute for cow's milk; tannins and dyes extracted from barks
* Pouteria caimito *(Ruiz & Pav.) Radlk*. (*= *Lucuma lasiocarpa* (Mart.) A.DC.)	abiurana	85	Wood, fruit	Native	General construction, food
Solanaceae					
* Capsicum annuum* L. (= *Capsicum frutescens* L.)	pimenta-de-caiena	98	Not mentioned	Cultivated	Spice
Styracaceae					
* Styrax benzoin* var. *benzoin* Dryand. (= *Benzoin officinale* Hayne.)	benjoim	107	Balm	Exotic	Not mentioned
Zingiberaceae					
* Aframomum melegueta* K.Schum. (= *Amomum grana-paradisi* L.)	malagueta	98	Not mentioned	Exotic	Spice
* Zingiber officinale* Roscoe (= *Amomum zinziba* Hill)	gengibre	98	Rhizome	Cultivated	Spice, beverage (ginger beer)

*Unrecognized name/spelling cited exactly in the book

**According to the 3rd edition (1899)

^a^Updated botanical names (in parenthesis = terminology as originally described by Santa-Anna Nery), ^b^Non-native to the Amazon, ^c^Endemic in Northeast and Southeast, ^d^Except in North Brazil, ^e^Northeast Brazil, ^f^Endemic in the sea coast

In a recent work by Pironon et al. [163], it is affirmed that there are currently 35,687 utilized plants in the world for human, vertebrate, and invertebrate food, materials (e.g., wood, fiber), fuels, social uses (e.g., narcotics, ritual, religious uses), poisons, medicines, environmental uses (e.g., intercrops, windbreaks, ornamentals), and gene sources (e.g., crop wild relatives). Some decades ago, Bennett [164] asked how ethnobotanists can help protect tropical forests and preserve the lives and traditional knowledge associated with Amazonia's indigenous peoples. The answer was that, first, they can identify native plant resources. That is the reason documenting and understanding the diversity and distribution of plant species used by humans is crucial to implementing conservation strategies and developing plant-based solutions to address global societal challenges such as hunger, diseases, and climate change [163].

In this context, historical ethnobotany emerges as a prominent science, which deals with the study of human-plant relationships in the past, primarily using the analysis of written historical documents such as publications, manuscripts, official records, and prescriptions, as well as iconographic sources and voucher specimens in herbaria [165–167]. Traditional knowledge about plant use and effectiveness contributes, for example, to the dissemination of therapeutic benefits, validating the information accumulated over centuries [168].

In this work we assessed such type of historical information and our results confirm that the book “Le Pays des Amazones” [32] is one of the sources containing valuable information about traditional uses of Amazonian biodiversity in the nineteenth century. Based on documents and reports from naturalists and his youthful memories and own traveling experiences, the Baron de Santa-Anna Nery shows to readers the huge potential of plant biodiversity. Santa-Anna Nery was in Paris when he wrote his main works about the Amazon, so many of his references were in his own past, in his memories, and in the indigenous objects he kept in his private collection. His experience among indigenous tribes during childhood, accompanying catechetical projects, was also important for his work. However, he undertook three journeys to the Amazon Basin regions from 1882 to 1887, interacting with the residents of the area, including indigenous peoples who became his informants [33, 169].

Starting from an exploratory perspective that promoted the idea that progress would be achieved through the manipulation and cultivation of nature, in line with the thoughts idealized by Count Buffon a century earlier [170], Baron de Santa-Anna Nery portrays the Amazon rainforest as "*the land of rubber, the legendary El Dorado, the virgin lands awaiting the sowing of civilization. Its fauna is infinite; its flora, inexhaustible; its mineral wealth, mysterious*". For Santa-Anna Nery [35], the Amazon Valley was destined to become a significant agricultural center if Europeans were willing to emigrate to the region: "*Soon we will see the immense forests partially cleared, the banks of the great rivers cleansed, the interior plains transformed into cultivated fields. All this vastness, almost deserted today, will give way to productive work*".

However, Santa-Anna Nery [35] warned of the need to study the Amazon before European exploration: "*Civilization is sometimes synonymous with destruction. Man, in mastering Nature, mutilates it. He exterminates to reign. Instead of subjugating animals for his service, he kills them indiscriminately, without thinking of adapting them for domestic use. Instead of extracting from the forests what is necessary for his needs of food, shelter, furniture, navigation, medication, he fells randomly, with prodigal recklessness, and destroys, for the future, precious sources of well-being. It would be desirable for the naturalists and scientists to pass through the virgin lands before the settler, to establish the region's resources and ensure their conservation*".

To better understand the propagandist intentions of Baron de Santa-Anna Nery in attracting European immigrants to the Amazon, it is important to demographically contextualize the Amazon region at the end of the nineteenth century. According to the census conducted at the end of 1890, the state of Amazonas, Brazil, had 147,115 inhabitants, representing a population density of about 0.08 inhabitants per square kilometer [35]. The Amazon, in this geographic scope comprising the states of Pará and Amazonas, was still considered wild, indigenous, and sparsely populated at this time—an erroneous and prejudiced view, according to Baron de Santa-Anna Nery himself [33]. The population of the state of Amazonas, according to Santa-Anna Nery, consisted of three main elements: Brazilians of all descents, catechized indigenous people, and foreigners from various origins, not counting the indigenous people who remained in a wild state [35].

From the cultural and social legacy of the Portuguese, who imposed their culture, ways of living, and modes of production on the indigenous people, along with the Northeastern Brazilians who came to work in the rubber tree (*Hevea brasiliensis* (Willd. ex A.Juss.) Müll.Arg.) plantations, transformations occurred marked by intense and diverse cultural exchanges [171]. The indigenous people were affected by diseases brought by the Europeans, such as smallpox, which proved fatal, and they succumbed to addictions like alcohol. Santa-Anna Nery also highlights that slave labor was incipient in the Amazon region, with at most a thousand enslaved Africans, and its abolition occurred in 1884, four years before the total abolition of slavery in Brazil. Rubber extraction, for example, was carried out mainly through the exploitation of the labor of indigenous people (who, according to a decree from 1757, could not be enslaved) and Northeastern migrants, especially from the state of Ceará, Brazil. It is in this context, from the second half of the nineteenth century, that Brazil sought to attract immigrants to occupy the regions far from major population centers. Despite the amount spent on the migration campaign, between 1855 and 1882, the country received about 500,000 immigrants, who mostly headed south, mainly Italians and Germans, while the north remained uninhabited [35].

Inserted into this demographic context of the Amazon highlighted by Santa-Anna Nery, he also presents in his book a survey regarding indigenous ethnicities from 1768 until the end of the nineteenth century (1899). He provides the names, place of origin identified by the river where they lived, and characteristics of 373 different ethnicities that existed in the state of Amazonas [35]. Of all the social elements in the Amazon, the indigenous population underwent the most significant cultural dismantling. Through interactions with Portuguese colonizers, the traditional knowledge of the indigenous people began to incorporate practices and knowledge considered civilized, mainly derived from European folk medicine. In addition to the Europeans, the indigenous people of the Amazon also engaged in the intense interactions with Northeastern Brazilians, introducing elements of African traditions inherited from enslaved Africans [171]. A valuable ethnobotanical contribution of the Baron, in this way, is the citation of vernacular names in the original language, with most of these names having indigenous origins, followed by Portuguese names.

Regarding botanical biodiversity, Baron de Santa Anna-Nery, drawing on his own knowledge and documents from naturalists of the eighteenth and nineteenth centuries, presented various possibilities for using Amazonian plants. The choice of plant species was driven by two main aspects: (a) To highlight the varieties of native natural resources that immigrants would find in the Amazon, demonstrating that there would be no shortage of raw materials for their daily needs, ranging from food to woods for building houses, substitutes for some plants used in Europe, such as guarana replacing coffee, appealing to the exoticism and vegetal abundance of the forest. There is an appreciation for regional plants and derived products, such as fruits, beverages, and stimulant plants, aiming to attract visitors eager to experience exotic products; (b) from an economic perspective, it mentioned native plants that could foster agricultural practices or generate profits through their exploitation [33].

In this sense, Santa-Anna Nery mentions in his book several plant species that were already well-known internationally at the end of the nineteenth century, precisely because they were involved in highly lucrative economic cycles, aiming to supply raw materials for export [172], such as the rubber tree (*Hevea brasiliensis*), copaiba (*Copaifera* spp.), ipecac (*Carapichea ipecacuanha* (Brot.) L. Andersson), “breu” oleoresin (*Protium glabrum* (Rose) Engl.), coca (*Erythroxylon coca* Lam.), “salsaparrilha” (*Smilax* spp.) and Brazil nut (*Bertholletia excelsa* Bonpl.), many of them formerly included in official medical compendia, such as the European Pharmacopoeias [3, 173–175]. Other plants were internationally recognized as foods and beverages, like guarana (*Paullinia cupana* Kunth), cashew (*Anacardium occidentale* L.), cacao (*Theobroma* spp.), manioc (*Manihot esculenta* Crantz.), pineapple (*Ananas comosus* (L.) Merr.), vanilla (*Vanilla planifolia* Andrews) and “pupunha” (*Bactris gasipaes* var. *gasipaes* Kunth), and others were extensively explored also for dyeing, such as *Bixa orellana* L. [173], and perfumery and carpentry, such as *Dipteryx odorata* (Aubl.) Forsyth f. and *Cedrela odorata* L., respectively [172].

Many plants cited in Santa-Anna Nery's book have been continuously compiled in various ethnobotanical studies in the Amazon since the end of the twentieth century: *Acmella oleracea* (L.) R.K. Jansen (medicine)*, Anacardium occidentale* (food), *Anadenanthera peregrina* (L.) Speg. (not mentioned)*, Ananas comosus* (food, medicine), *Astrocaryum vulgare* Mart. (construction, objects), *Bertholletia excelsa* (food, medicine, construction), *Bixa orellana* (dye, food, medicine), *Bowdichia virgilioides* Kunth (not mentioned)*, Brunfelsia uniflora* (Pohl) D.Don (medicine), *Calophyllum brasiliense* Cambess. (not mentioned)*, Capsicum annuum* L. (medicine), *Carica papaya* L. (food, medicine), *Cedrela odorata* (medicine), *Copaifera* spp. (medicine)*, Dioscorea* sp. (food), *Dipteryx odorata* (medicine), *Elaeis oleifera* (Kunth) Cortés (food, medicine, construction), *Euterpe oleracea* Mart. (food), *Genipa americana* L. (dye, construction, food, medicine), *Humiria balsamifera* (Mart.) Cuatrec. (not mentioned)*, Hymenaea courbaril* L. (not mentioned)*, Inga* spp. (food, forage, fuel, medicine), *Jacaranda copaia* (Aubl.) D.Don (medicine), *Leopoldinia piassaba* Wallace (food, medicine, construction), *Manicaria saccifera* Gaertn. (food, medicine, construction), *Manihot esculenta* (food, ritual/mythical, medicine), *Mauritia flexuosa* L.f. (food), *Musa x paradisiaca* L. (medicine), *Oenocarpus bacaba* Mart. (food, medicine, construction), *Oenocarpus bataua* Mart. (food, medicine, construction), *Paullinia cupana* (medicine)*, Paullinia pinnata* L. (toxic), *Pradosia lactescens* (Vell.) Radlk) (not mentioned)*, Psidium guajava* L. (food, medicine), *Theobroma cacao* L. (food, medicine) and *Vouacapoua americana* Aubl. (not mentioned) [164, 176–181].

It is interesting to observe how many plants presented by Santa-Anna Nery continue to be traditionally used by Amazonian communities, even after almost 150 years since the book was published. These plants demonstrate resilience over time, persisting despite the availability of new and more effective medicines, improvements in health care, changes in epidemiology, and advancements in sanitary conditions. The resilience of local ecological systems is crucial for preserving local identity and culture [182].

Nevertheless, the majority of plants cited in Table 1 or 2 are restricted to a local Amazonian knowledge, not being included in medicinal or useful Brazilian plants guides. Only 23 non-native plants were cited in the inventory, such species introduced in Brazil since the sixteenth century by the Portuguese due to their economic, edible or medicinal potential [183]. Exotic species such as *Tamarindus indica* L., *Zingiber officinale* Roscoe, *Styrax benzoin* var. *benzoin* Dryand. and *Cinnamomum* sp. are native to African and Asian continents, while *Smilax glauca* Walter, *Lecythis ollaria* L., *Protium glabrum*, *Psidium guajava*, *Carica papaya* and *Ipomoea batatas* (L.) Lam. are native from America, mainly Central America. Some of these plants were important raw material and foods and were cultivated or naturalized for economic exploitation. That is the reason it is so important and strategic to access the potential of Amazonian native useful plants in Brazil. The Brazilian native plants are still very poorly known and their potential is still insufficiently exploited, which is further aggravated by the fact that these species are very often distributed in areas subjected to intense human action and therefore under severe threat of extinction [184]. The sustainable use of native biodiversity for bioeconomic purposes is one of the aspects of technological innovation in the twenty-first century. Sustainable management of resources from the Amazonian flora allows for income generation for small producers, providing raw materials for the production of genuinely local products such as cosmetics, phytotherapeutic medicines, traditional phytotherapeutic products, agricultural products, and more. Traditional knowledge related to plant species of industrial interest can be utilized for the development of products and processes that should benefit communities through fair and equitable benefit sharing [185].

Santa-Anna Nery predominantly highlighted in his work many native edible plants as well as those used in civil and naval construction. Large and imposing trees, such as "maçaranduba" (*Manilkara bidentata* (A.DC.) A. Chev. subsp. *bidentata*) and "guarabu" or "pau-roxo" (*Peltogyne* sp.), are described with heights exceeding 20 m and trunks measuring 1.20–2 m in diameter. The former is employed in the construction of railways and ship wedges, while the latter is esteemed for its high-quality wood. According to Santa-Anna Nery [32], “maçaranduba” was one of the most precious forest products in the Amazon at that time.

With descriptions like these, the Baron seeks to draw the attention of Europeans to what he deems "normal exploration" of the forest, that is, the use of natural resources for the everyday and economic needs of the community. In his words: "*Wood is already beginning to be scarce in old Europe, deprived of its prehistoric forests; the time is not far off when we will be forced to seek new forest reserves in the new world. The Amazon holds in reserve, for centuries, a supply of wood capable of sustaining all local and foreign industries*" [35].

In "Le Pays des Amazones," we find descriptions of various preparations based on native edible plants. For example, the cassava root (*Manihot esculenta*) is used in the production of both white and yellow flours, often as a substitute for bread, or in the preparation of “cauim,” an indigenous alcoholic beverage. This root is considered the staple of the population's diet and is commonly used in porridges and soups. “Tucupi'' is also mentioned, a liquid extracted from cassava, which is grated and compressed in the “tipiti,” an elastic tube made from the stalks of plants like “jacitara” (*Desmoncus* sp.) or “guarumá” (*Ischnosiphon arouma* (Aubl.) Körn.). “Tucupi,” when raw, was considered a violent plant poison containing cyanic acid as the active principle, although it is safe for consumption after boiling, as cyanic acid is volatile [32]. Indeed, cyanogenic glycosides are found in high percentages in cassava roots and leaves, such as linamarin and lotaustralin. These active constituents have neurotoxic and neurological effects because, after undergoing hydrolysis, they release cyanide derivatives. Cyanogenic compounds need to be removed by boiling, peeling, fermenting, and cooking the plant, resulting in a loss of up to 70% of these toxic substances [186].

The use of plants such as *Serjania ferruginea* (Lindl.) Mabb., *Paullinia pinnata*, and *Magonia* sp., known by the generic term "timbós" and utilized by indigenous people for fishing, is also described in the work: "*The timbó was crushed, and the juice obtained was poured into a shallow and calm lake or river. After a few hours, the intoxicated fish would appear on the water's surface, and it was only a matter of collecting them in a boat; the small fish were disregarded*" [32]. For instance, plants of the genus *Serjania* (e.g., *S. lethalis* A.St.-Hil.) contain saponins called serjanosides, with ichthyotoxic properties [187], and some species (e.g., *S. tenuifolia* Radlk., *S. ferruginea*) are still used today by traditional peoples of the Amazon [188–190].

Due to its importance in the late nineteenth century, the latex extracted from the rubber tree (*Hevea* spp.) has a chapter exclusively dedicated to its historical, social, and economic description. The first rubber boom lasted from 1880 until 1913, while the second boom resumed during World War II (1939–1945) [191].

According to Santa-Anna Nery [35], by the end of the 1890s, the annual international rubber consumption was around 50,000 tons. Rubber latex from the rubber tree was exported in its raw state, and abroad, the raw material was transformed and resold at higher prices to Brazil. Due to the significant interest at that time, especially driven by the automotive and bicycle industries that used latex for tire manufacturing, several European metropolises attempted to acclimate the rubber tree in their warm-climate colonies.

One well-known case is that of the British explorer Henry Alexander Wickham, who, at the service of the Kew Royal Botanical Garden, smuggled more than 70,000 *Hevea* seeds in 1876, with the purpose of the British crown cultivating them in Asian colonies. The 1910s were marked by rubber production in the East, notably English rubber produced in Malaysia, which timidly produced three tons in 1900, compared to over 26,000 tons produced in the Brazilian Amazon. In 1913, it began producing 47,000 tons compared to 38,000 tons of Brazilian rubber, marking the year of the break in the Brazilian monopoly on rubber export in favor of international production. By 1921, rubber plantations in the East were producing 1.5 million tons of rubber, compared to only 20,000 tons from the Amazon [192].

The nineteenth century was marked by the advancement of chemistry, with the isolation of the first molecule of natural origin, morphine, by the German pharmacist Friedrich Setürner in 1806 from opium (*Papaver somniferum* L.). This event revolutionized medicine, as the treatment of diseases using isolated substances (drugs) led to more effective and safer outcomes, with the standardization of dosages. Since then, throughout that century, the interest of pharmacists and chemists in isolating new molecules that could be used in therapy led to the isolation of numerous other drugs of plant origin, such as quinine, caffeine, atropine, and digoxin [193]. Baron de Santa-Anna Nery gives great prominence to native medicinal plants in "Le Pays des Amazones," once again focused on promoting Amazonian natural resources, but mainly emphasizing the idea of developing the industrialization process allied to the processing and rational exploitation of forest products, along with agricultural development to achieve an "economic revolution" [33].

Based on a documentary analysis of other naturalists who described the Amazon, Santa-Anna Nery accurately indicates the medicinal properties of various plants, while some species are simply classified as "medicinal" (undefined), probably based on his own memories regarding the therapeutic potential of plants he encountered when he was younger but did not find descriptions in the works of the naturalists he studied. In this study, it was possible to deepen the knowledge about the medicinal use of species through pharmacological studies, establishing a connection with the traditional usage information from the late nineteenth century to the pharmacological properties studied since then until the present day.

Pharmacological studies corroborate the traditional uses for many related medicinal plants: astringent plants such *Bowdichia virgilioides* [64], *Krameria argentea* Mart. ex Spreng. [84–86], and *Psidium guajava* [128]; laxative/purgative plants such as *Tamarindus indica* [79–81]; vermifuge plants such as *Carica papaya* [52], *Ficus gomelleira* Kunth & C.D.Bouché [30, 31] and *Spigelia anthelmia* L. [115, 116]; bitter tonic and febrifuge plants such as *Carapa procera* DC. [106] and *Cedrela odorata* [109]; expectorant, emetic, and amoebicidal properties of *Carapichea ipecacuanha* [131–133]; antivenom properties of *Brunfelsia uniflora* [154]; anesthetic, analgesic, anti-inflammatory and anti-scorbutic actions of *Acmella oleracea* [41–43]; stimulant activity of *Paullinia cupana* [141–143] and *Erythroxylum coca* [62, 63].

The indigenous procedure for preparing guaraná (*P. cupana*) for stimulant purposes is described in detail: “*Its seeds are used to create a stimulating beverage by lightly roasting the beans, after drying them in the sun, and reducing them, with the addition of a small amount of water, to a paste to which whole or crushed seeds are added as desired. The guaraná paste is exported in the form of very hard sticks, with a reddish-brown color. The inhabitants of the region prepare the beverage by grating the guaraná with the dried tongue of the "pirarucu" fish (*Arapaima gigas*)*” [32]. The procedure of the Sateré-Maué indigenous people, regarding the preparation of guaraná, is well-documented in scientific literature [194, 195].

No pharmacological studies were found to corroborate the emetic, laxative, and antisyphilitic activities of *Jacaranda copaia*, laxative action of *Tabebuia insignis* (Miq.) Sandwith. nor even *Tecoma* spp., astringent properties of *Couepia* sp. nor treatment of skin infections of *Vateria* sp.

Although many plant species cited in the book are only mentioned as “medicinal,” currently there are many studies confirming their pharmacological properties: hypoglycemic and antioxidant activities of *Cassia grandis* L.f. [69, 70]; anti-inflammatory, analgesic, wound healing, antimicrobial, anticariogenic and antiparasitic activities of *Copaifera* spp. [71–77]; anti-inflammatory and antiplatelet aggregation of *Dipteryx odorata* [78]; antifungal and anti-inflammatory potential of *Aniba puchury-minor* (Mart.) Mez [89]; antifungal and anticoagulant activities of *Ocotea sassafras* (Meisn.) Mez. [90, 91]; wound healing activity and Selenium poisoning potential of *Lecythis ollaria* [92–94]; antioxidant, anti-inflammatory and antialopecia activities, and neuroprotective, cardioprotective, hepatoprotective and nephroprotective potential of *Theobroma cacao* [100–105]; gastroprotective, antimicrobial, wound healing, antinociceptive and anti-inflammatory activities of *Virola bicuhyba* (Schott) Warb. [117–120]; antiplatelet, anti-inflammatory, neuroprotective, antioxidant, anti-glycant, antithrombotic, anticonvulsant and trypanocidal activities of *Genipa americana* [135–140]; anti-wrinkling and anti-melanogenic of *Pradosia lactescens* [148]; and nephroprotective, anti-inflammatory, cytotoxic, antioxidant, antihyperalgesia and antiasthmatic activities of *Zingiber officinale* [158–162].

Based on our results, we understand historical ethnobotany as a tool for biodiversity protection, aiming to understand the relationship and use in the past, as well as the development over time and the result of this use in the current context. Therefore, by learning from past experiences, it comprehends the present and facilitates the creation of sustainable and protective methods and solutions for the future. It demonstrates which uses and therapeutic indications are suitable for investigation, particularly regarding native plants, valuing and documenting the traditional knowledge of cultures affected by cultural erosion [196, 197]. In this sense, pharmacological investigations are important to ascertain the presence of active principles and compounds that have a specific therapeutic action, correlating traditional knowledge with scientific knowledge, thus directing research toward the possible development of a new drug, herbal medicine, or traditional herbal product [196]. The importance of this type of study is also due to the increase in deforestation in recent years, which contributes to genetic and cultural erosion of plants in Brazil, favoring the introduction of monoculture and exotic plants, consequently reducing biodiversity and the traditional use of native plants in the region [12].

More than a century later, Baron de Santa-Anna Nery [35] was right about a fact: "*The Amazonian flora is still not entirely known; much is still lacking. A large number of species have not been taxonomically classified or described*". Taxonomic data reveals that over 14,000 species are cataloged, with almost half of these being trees [198, 199]. However, it is estimated that the Amazon Basin region has over 50,000 plant species [200], with the majority remaining unknown.

## Conclusion

The book "Le Pays des Amazones" was written and published during a period when very little was known about the Amazon region. When we analyze this work from a timeless perspective, with a particular focus on historical ethnobotany, we gain insights into the prevalent medical concerns of the time and the plant remedies employed to address these conditions. Furthermore, it becomes apparent that many of the medicinal plants documented in the book have since been the subject of pharmacological studies that confirm their therapeutic properties, while many other species still remain to be investigated. Besides medicinal plants, the analysis of this work inventoried various traditional uses of plant species, providing insights into understanding historical, social, and economic aspects of the late nineteenth century. Uncovering the historical ethnobotanical knowledge within "Le Pays des Amazones" is essential for preserving and sharing the history and contributions of Baron de Santa-Anna-Nery, whose legacy has somewhat faded in the broader context of Brazilian culture and literature.

## Data Availability

All data generated or analyzed during this study are included in this published article.

## Abbreviations

AM: Amazonas
CNS: Central nervous system
JBRJ: Jardim Botânico do Rio de Janeiro
PA: Pará
RJ: Rio de Janeiro
UFOPA: Universidade Federal do Oeste do Pará
UFRJ: Universidade Federal do Rio de Janeiro

## References

[1] Brandão MGL, Zanetti NNS, Oliveira P, Grael CFF, Santos ACP, Monte-Mór RLM (2008). Brazilian medicinal plants described by 19th century European naturalists and in the Official Pharmacopoeia. J Ethnopharmacol.

[2] Losada JZ, Puig-Samper MA, Domingues HMB (2014). Um álbum para o Imperador: a Comissão científica do Pacífico e o Brasil.

[3] Sanjad N, Pataca E, Santos RRN (2021). Knowledge and Circulation of Plants: Unveiling the participation of Amazonian indigenous peoples in the construction of eighteenth and nineteenth century Botany. J Hist Sci Technol.

[4] Cancela F (2021). A flora da antiga capitania de Porto Seguro na viagem de Wied-Neuwied, 1815–1817: prática científica, inventário naturalista e colaboração indígena. Hist Cienc Saúde-Man.

[5] Breitbach UB, Niehues M, Lopes NP, Faria JEQ, Brandão MGL (2013). Amazonian Brazilian medicinal plants described by C.F.P. von Martius in the 19th century. J Ethnopharmacol.

[6] Brandão MGL, Pignal M, Romaniuc S, Grael CFF, Fagg CW (2012). Useful Brazilian plants listed in the field books of the French naturalist Auguste de Saint-Hilaire (1779–1853). J Ethnopharmacol.

[7] Fagg CW, Lughadha EN, Milliken W, Hind DJN, Brandão MGL (2015). Useful Brazilian plants listed in the manuscripts and publications of the Scottish medic and naturalist George Gardner (1812–1849). J Ethnopharmacol.

[8] Alves JJA (2011). A natureza e a cultura no compasso de um naturalista do século XIX: Wallace e a Amazônia. Hist Cienc Saúde-Man.

[9] Ferreira RS (2004). Henry Walter Bates: um viajante naturalista na Amazônia e o processo de transferência da informação. Ci Inf.

[10] Alves JJA (2022). Motivações e condições para a viagem de um naturalista na Amazônia: Henry Bates. Cadernos de História da Ciência.

[11] Santos-Fonseca DJ, Coelho-Ferreira M, Fonseca-Kruel VS (2019). Useful plants referenced by the naturalist Richard Spruce in the 19th century in the state of Pará, Brazil. Acta Bot Brasilica.

[12] Brandão MGL (2010). Plantas úteis nativas do Brasil na obra dos naturalistas. Hortic Bras.

[13] Pigafetta A. Relazione del primo viaggio intorno al mondo: a cura di Camillo Manfroni. Milano: Istituto Editoriale Italiano; 1956. https://www.liberliber.it/online/autori/autori-p/antonio-pigafetta/relazione-del-primo-viaggio-intorno-al-mondo/. Accessed 19 Jan 2024.

[14] Hernandez F. Quatro libros. De la naturaleza, y virtudes de las plantas, y animales que estan receuidos en el vso de medicina en la Nueua España, y la methodo, y correccion, y preparacion, que para administrallas se requiere con lo que el doctor Francisco Hernandez escriuio en lengua latina: Muy util para todo genero de gente q[ue] vive en esta[n]cias y pueblos, de no ay medicos, ni botica. Traductor al español: Francisco Ximenez. Mexico: Casa de viuda de Diego Lopez Daualos; 1615. https://www.biodiversitylibrary.org/item/136370#page/7/mode/1up. Accessed 19 Jan 2024.

[15] Monardes NB. De simplicibus medicamentis ex occidentali India delatis quorum in medicina usus est. Antwerp: Ex officina Christophori Plantini; 1574. https://www.biodiversitylibrary.org/item/36580#page/1/mode/1up. Accessed 19 Jan 2024.

[16] Sessé M. Flora Mexicana. Mexico: I. Escalante; 1887. https://www.biodiversitylibrary.org/item/120013#page/2/mode/1up. Accessed 19 Jan 2024.

[17] Sessé M. Plantae Novae Hispaniae. Mexico: Oficina tip. de la Secretaría de fomento; 1893. https://www.biodiversitylibrary.org/item/120055#page/1/mode/1up. Accessed 19 Jan 2024.

[18] Van Andel T, Veldman S, Maas P, Thijsse G, Eurlings M (2012). The forgotten Hermann herbarium: a 17th century collection of useful plants from Suriname. Taxon.

[19] Sloane H. A voyage to the islands Madera, Barbados, Nieves, S. Christophers and Jamaica. 2 volumes. London: B.M.; 1707–1725. https://www.biodiversitylibrary.org/bibliography/153795. Accessed 19 Jan 2024.

[20] Ruiz H, Pavón J. Flora Peruviana, et Chilensis, sive, Descriptiones et cones plantarum Peruvianarum, et Chilensium, secundum systema Linnaeanum digestae, cum characteribus plurium generum evulgatorum reformatis, vol. 3. Madrid: Typis Gabrielis de Sancha; 1798–1802. https://www.biodiversitylibrary.org/bibliography/814. Accessed 19 Jan 2024.

[21] Britannica. The Editors of Encyclopaedia. "Joseph de Jussieu". Encyclopedia Britannica. https://www.britannica.com/biography/Joseph-de-Jussieu. Accessed 19 Jan 2024.

[22] Mutis y Bosio JC. Flora de la Real Expedición Botánica del Nuevo Reino de Granada: (1783–1816). 50 volumes. Madrid: Ediciones Cultura Hispánica; 1954–2001. https://bibdigital.rjb.csic.es/records/item/15841-redirection. Accessed 19 Jan 2024.

[23] Plumier C. Plantarum Americanarum. 8 volumes. Amstelaedamum: Lugdunum Batavorum; 1755–1760. https://bibdigital.rjb.csic.es/records/item/14938-redirection. Accessed 19 Jan 2024.

[24] Aublet JCBF. Histoire des plantes de la Guiane Françoise. 4 volumes. Londres/Paris: Chez Pierre-Francois Didot jeune, Libraire de la Faculté de Médecine, Quai des Augustins; 1775. https://bibdigital.rjb.csic.es/records/item/13937-redirection. Accessed 19 Jan 2024.

[25] La Condamine CM. Relation abrégée d’un voyage fait dans l’interieur de l’Amérique méridionale. Paris: chez la veuve Pissot, quay de Conti, a la Croix d'Or; 1745. https://www.biodiversitylibrary.org/item/182549#page/7/mode/1up. Accessed 19 Jan 2024.

[26] Baratto LC (2022). Useful plants described in the Plantes Équinoxiales (1805–1817) by Alexander von Humboldt and Aimé Bonpland. Bot Lett..

[27] Mariath F, Baratto LC (2023). Female naturalists and the patterns of suppression of women scientists in history: the example of Maria Sibylla Merian and her contributions about useful plants. J Ethnobiol Ethnomedicine.

[28] Merian MS. Metamorphosis Insectorum Surinamensium. Amsterdam: Voor den auteur, als ook by G. Valck; 1705. https://www.biodiversitylibrary.org/bibliography/63607. Accessed 19 Jan 2024.

[29] Fontes E, Aragón LE (2009). A Imigração e mercado de trabalho na Amazônia do fim do século XIX: o caso dos portugueses em Belém do Pará. Migração Internacional na Pan-Amazônia.

[30] Carneiro JPJA (2013). O último propagandista do Império: o “barão” de Santa-Anna Nery (1848–1901) e a divulgação do Brasil na Europa.

[31] Lima MG. A Trajetória de Sant’Anna Nery: Um mediador entre o Brasil e a França. In: Anais XIV Congresso Internacional ABRALIC. 2015. https://abralic.org.br/anais-artigos/?id=957. Accessed 19 Jan 2024.

[32] Santa-Anna Nery FJ. Le Pays des Amazones: L’El-dorado Les Terres a Caoutchouc. 1st ed. Paris: Bibliotèque de deux-mondes; 1885. https://digital.bbm.usp.br/handle/bbm/285. Accessed 01 Nov 2023.

[33] Coelho ACA. Santa-Anna Nery: um propagandista “voluntário” da Amazônia (1883–1901). Belém: Universidade Federal do Pará; 2007. https://repositorio.ufpa.br/jspui/handle/2011/4194. Accessed 17 Dec 2023.

[34] Granger S (2019). O contestado Franco-Brasileiro: desafios e consequências de um conflito esquecido entre a França e o Brasil na Amazônia. Revista Cantareira.

[35] Santa-Anna Nery FJ. O país das Amazonas. 3rd ed. Brasília: Senado Federal; 2018. https://www2.senado.leg.br/bdsf/handle/id/574200. Accessed 01 Apr 2022.

[36] Santa-Anna Nery FJ. Le Pays des Amazones: L’El-Dorado, les terres a caoutchouc. 3rd ed. Paris: Libraire Guillaumin; 1899. https://www.biodiversitylibrary.org/bibliography/36485. Accessed 01 Nov 2023.

[37] Moro MF, Souza VC, Oliveira-Filho AT, Queiroz LP, Fraga CN, Rodal MJN, Araujo FS, Martins FR (2012). Alienígenas na sala: o que fazer com espécies exóticas em trabalhos de taxonomia, florística e fitossociologia?. Acta Bot Brasilica.

[38] Suffredini IB, Paciencia MLB, Varella AD, Younes RN (2007). In vitro cytotoxic activity of Brazilian plant extracts against human lung, colon and CNS solid cancers and leukemia. Fitoterapia.

[39] Rosa MN, Silva LRV, Longato GB, Evangelista AF, Gomes INF, Alves ALV, de Oliveira BG, Pinto FE, Romão W, de Rezende AR, Araújo AAC, Oliveira LSFM, de Souza AA, Oliveira SC, de Ribeiro RIM, Silva VAO, Reis RM (2021). Bioprospecting of natural compounds from Brazilian cerrado biome plants in human cervical cancer cell lines. Int J Mol Sci.

[40] Mendes RF, Pinto NC, da Silva JM, da Silva JB, dos Hermisdorf RCS, Fabri RL, Chedier LM, Scio E (2017). The essential oil from the fruits of the Brazilian spice Xylopia sericea A St-Hil presents expressive in-vitro antibacterial and antioxidant activity. J Pharm Pharmacol.

[41] Evans WC (2009). Trease and Evans’ Pharmacognosy.

[42] Boonen J, Baert B, Burvenich C, Blondeel P, de Saeger S, Spiegeleer B (2010). LC–MS profiling of N-alkylamides in *Spilanthes acmella* extract and the transmucosal behaviour of its main bio-active spilanthol. J Pharmaceut Biomed.

[43] Wu LC, Fan NC, Lin MH, Chu IR, Huang SJ, Hu CY, Han SY (2008). Anti-inflammatory effect of spilanthol from *Spilanthes acmella* on murine macrophage by down-regulating LPS-induced inflammatory mediators. J Agr Food Chem.

[44] Maria-Ferreira D, da Silva LM, Mendes DAGB, Cabrini DA, Nascimento AM, Iacomini M, Cipriani TR, Santos ARS, Maria W, Baggio CH (2014). Rhamnogalacturonan from *Acmella oleracea* (L.) R.K. Jansen: Gastroprotective and Ulcer Healing Properties in Rats. PLoS ONE.

[45] Gerbino A, Schena G, Milano S, Milella L, Barbosa AF, Armentano F, Procino G, Svelto M, Carmosino M (2016). Spilanthol from *Acmella oleracea* lowers the intracellular levels of cAMP impairing NKCC2 phosphorylation and water channel AQP2 membrane expression in mouse kidney. PLoS ONE.

[46] Houël E, Ginouves M, Azas N, Bourreau E, Eparvier V, Hutter S, Knittel-Obrecht A, Jahn-Oyac A, Prévot G, Villa P, Vonthron-Sénécheau C, Odonne G (2022). Treating leishmaniasis in Amazonia, part 2: Multi-target evaluation of widely used plants to understand medicinal practices. J Ethnopharmacol.

[47] Céline V, Adriana P, Eric D, Joaquina A, Yannick E, Augusto LF, Rosario R, Dionicia G, Michel S, Denis C, Geneviève B (2009). Medicinal plants from the Yanesha (Peru): evaluation of the leishmanicidal and antimalarial activity of selected extracts. J Ethnopharmacol.

[48] Ferraz-Filha ZS, Ferrari FC, de Araújo PM, Bernardes ACFPF, Saúde-Guimarães DA (2017). Effects of the aqueous extract from *Tabebuia roseoalba* and phenolic acids on hyperuricemia and inflammation. Evid-Based Compl Alt..

[49] Barrios-Nolasco A, Domínguez-López A, Miliar-García Á, Cornejo-Garrido J, Jaramillo-Flores ME (2023). Anti-inflammatory effect of ethanolic extract from *Tabebuia rosea* (Bertol.) DC., quercetin, and Anti-obesity drugs in adipose tissue in wistar rats with diet-induced obesity. Molecules.

[50] da Silva SMA, da Silvaneto GJ, do Nascimento IRC, Viana MDM, de Lima AA, Bezerra PHS, de Bastos MLA, Moreira MSA, Campesatto EA (2018). The antinociceptive effect of the leaves and flowers ethanolic extracts of *Tabebuia aurea* (Silva Manso) Benth & Hook. F. ex S. Moore. Braz Arch Biol Techn.

[51] Kameshwara S, Jothimaniv C, Senthilkum R, Thenmozhi S, Sundaragan R, Dhanalaksh M (2013). Acute toxicity study and faecal dropping capability of ethanolic extract of *Tecoma stans* in albino rats. Pharmacologia.

[52] Kermanshai R, McCarry BE, Rosenfeld J, Summers PS, Weretilnyk EA, Sorger GJ (2001). Benzyl isothiocyanate is the chief or sole anthelmintic in papaya seed extracts. Phytochemistry.

[53] Guzmán C, Villalobos N, Caltempa AO, Hernández M, Núñez G, Salazar J, Bobes RJ, Fragoso G, Sciutto E, Villarreal ML (2023). *In vitro* and *in vivo* cysticidal effects of *Carica papaya* cell suspensions. Infect Immun.

[54] Roy JR, Coimbatore S, Jayaraman S, Periyasamy V, Balaji T, Vijayamalathi M, Vishnu P (2022). Effect of *Carica papaya* on IRS-1/Akt signaling mechanisms in High-Fat-Diet–Streptozotocin-Induced type 2 diabetic experimental rats: a mechanistic approach. Nutrients.

[55] Mohd Abd Razak MR, Norahmad NA, Md Jelas NH, Afzan A, Mohmad Misnan N, Mat Ripen A, Thayan R, Zainol M, Syed Mohamed AF (2021). Immunomodulatory activities of *Carica papaya* L. leaf juice in a non-lethal, Symptomatic Dengue Mouse Model. Pathogens.

[56] Zunjar V, Dash RP, Jivrajani M, Trivedi B, Nivsarkar M (2016). Antithrombocytopenic activity of carpaine and alkaloidal extract of *Carica papaya* Linn. leaves in busulfan induced thrombocytopenic Wistar rats. J Ethnopharmacol.

[57] Berto A, Ribeiro AB, Sentandreu E, Souza NE, Mercadante AZ, Chisté RC, Fernandes E (2015). The seed of the Amazonian fruit *Couepia bracteosa* exhibits higher scavenging capacity against ROS and RNS than its shell and pulp extracts. Food Funct.

[58] Zuque ALF, Watanabe ES, Ferreira AMT, Arruda ALA, Resende UM, Bueno NR, Castilho RO (2004). Avaliação das atividades antioxidante, antimicrobiana e citotóxica de *Couepia grandiflora* Benth (Chrysobalanaceae). Rev Bras Farmacogn..

[59] Mirza MA, Hasan M, Ramesh S, Siddiqui MRH, Khan M, Shaik MR, Khan M (2023). *Vateria indica* (Linn) resin based ointment for the topical treatment of Radiation-Induced burns in cancer patients. J King Saud Univ Sci.

[60] Alshabi AM, Shaikh IA, Asdaq SMB (2022). The antiepileptic potential of *Vateria indica* Linn in experimental animal models: effect on brain GABA levels and molecular mechanisms. Saudi J Biol Sci.

[61] D’Souza JN, Nagaraja GK, Prabhu A, Navada KM, Kouser S, Manasa DJ (2022). AgVI and Ag/ZnOVI nanostructures from *Vateria indica* (L.) exert antioxidant, antidiabetic, anti-inflammatory and cytotoxic efficacy on triple negative breast cancer cells in vitro. Int J Pharm.

[62] Casikar V, Mujica E, Mongelli M, Aliaga J, Lopez N, Smith C, Bartholomew F (2010). Does chewing coca leaves influence physiology at high altitude?. Indian J Clin Biochem.

[63] Restrepo DA, Saenz E, Jara-Muñoz OA, Calixto-Botía IF, Rodríguez-Suárez S, Zuleta P, Chavez BG, Sanchez JA, D'Auria JC (2019). *Erythroxylum* in focus: an interdisciplinary review of an overlooked genus. Molecules.

[64] Agra IKR, Pires LLS, Carvalho PSM, Silva-Filho EA, Smaniotto S, Barreto E (2013). Evaluation of wound healing and antimicrobial properties of aqueous extract from *Bowdichia virgilioides* stem barks in mice. An Acad Bras Cienc.

[65] Somensi LB, Costa P, Boeing T, Mariano LNB, Gregório E, Silva ATM, Longo B, Locatelli C, Souza P, Magalhães CG, Duarte LP, Silva LM (2022). Lupeol stearate accelerates healing and prevents recurrence of gastric ulcer in rodents. Evid-Based Compl Al.

[66] Silva AC, dos Santos MP, de França SA, da Silva VC, da Silva LE, de Figueiredo US, Dall'Oglio EL, Júnior PT, Lopes CF, Baviera AM, Kawashita NH (2015). Acute and subchronic antihyperglycemic activities of *Bowdichia virgilioides* roots in non-diabetic and diabetic rats. J Intercult Ethnopharmacol.

[67] Silva JP, Rodarte RS, Calheiros AS, Souza CZ, Fabio C, Martins MA, Patrícia S, Valber S, Barreto E (2010). Antinociceptive activity of aqueous extract of *Bowdichia virgilioides* in mice. J Med Food.

[68] Thomazzi SM, Silva CB, Silveira DCR, Vasconcellos CLC, Lira AF, Cambui EVF, Antoniolli AR (2010). Antinociceptive and anti-inflammatory activities of *Bowdichia virgilioides* (sucupira). J Ethnopharmcol..

[69] Prada AL, Amado JRR, Keita H, Zapata EP, Carvalho H, Silva Lima EF, Pereira de Sousa T, Tavares Carvalho JC (2018). *Cassia grandis* fruit extract reduces the blood glucose level in alloxan-induced diabetic rats. Biomed Pharmacother.

[70] Prada AL, Keita H, Souza TP, Lima ES, Acho LDR, Silva MJA, Carvalho JCT, Amado JRR (2019). *Cassia grandis* Lf nanodispersion is a hypoglycemic product with a potent α-glucosidase and pancreatic lipase inhibitor effect. Saudi Pharm J.

[71] Alvarenga MOP, Bittencourt LO, Mendes PFS, Ribeiro JT, Lameira OA, Monteiro MC, Barboza CAG, Martins MD, Lima RR (2020). Safety and effectiveness of copaiba oleoresin (*C. reticulata Ducke*) on inflammation and tissue repair of oral wounds in rats. Int J Mol Sci.

[72] Alves JA, Abrão F, da Silva Moraes T, Damasceno JL, dos Santos Moraes MF, Sola Veneziani RC, Ambrósio SR, Bastos JK, Dantas Miranda ML, Gomes Martins CH (2020). Investigation of *Copaifera genus* as a new source of antimycobaterial agents. Future Sci OA.

[73] Santiago MB, Santos VCO, Teixeira SC, Silva NBS, Oliveira PF, Ozelin SD, Furtado RA, Tavares DC, Ambrósio SR, Veneziani RCS, Ferro EAVF, Bastos JK, Martins CHG (2023). Polyalthic acid from *Copaifera lucens* demonstrates anticariogenic and antiparasitic properties for safe use. Pharmaceuticals.

[74] Paiva LAF, Cunha KMA, Santos FA, Gramosa NV, Silveira ER, Rao VSN (2002). Investigation on the wound healing activity of oleo-resin from *Copaifera langsdorffi* in rats. Phytother Res.

[75] Paiva LAF, Gurgel LA, Silva RM, Tomé AR, Gramosa NV, Silveira ER, Santos FA, Rao VSN (2002). Anti-inflammatory effect of kaurenoic acid, a diterpene from *Copaifera langsdorffii* on acetic acid-induced colitis in rats. Vasc Pharmacol.

[76] Gelmini F, Beretta G, Anselmi C, Centini M, Magni P, Ruscica M, Cavalchini A, Maffei FR (2013). GC–MS profiling of the phytochemical constituents of the oleoresin from *Copaifera langsdorffii* Desf. and a preliminary in vivo evaluation of its antipsoriatic effect. Int J Pharm.

[77] Masson-Meyers D, Enwemeka CS, Violet B, Andrade TAM, Frade MA (2013). Topical treatment with *Copaifera langsdorffii* oleoresin improves wound healing in rats. Int J Phytomedicine.

[78] Wu L, Wang X, Xu W, Farzaneh F, Xu R (2009). The structure and pharmacological functions of coumarins and their derivatives. Curr Med Chem.

[79] Souza A, Aka KJ (2007). Spasmogenic effect of the aqueous extract of *Tamarindus indica* L. (Caesalpiniaceae) on the contractile activity of guinea-pig taenia coli. Afr J Tradit Complement Altern Med.

[80] Panthong A, Khonsung P, Kunanusorn P, Wongcome T, Pongsamart S (2008). The laxative effect of fresh pulp aqueous extracts of Thai Tamarind cultivars. Planta Med.

[81] Ali N, Shah SWA (2010). Spasmolytic activity of fruits of *Tamarindus indica* L. J Young Pharm.

[82] Shaimaa S, Aly SH, Shahira H, Allam A, Diab NH, Hassanein KM, Eissa RA, Eissa NG, Elsabahy M, Kamoun EA (2024). Electrospun *Tamarindus indica*-loaded antimicrobial PMMA/cellulose acetate/PEO nanofibrous scaffolds for accelerated wound healing: in-vitro and in-vivo assessments. Int J Biol Macromol.

[83] Adinath N, Mulla SI, Seth CS, Zabin K, Rahamathulla M, Ahmed MM, Farhana SA (2024). Phytochemical analysis, GC-MS profile and determination of antibacterial, antifungal, anti-inflammatory, antioxidant activities of peel and seeds extracts (chloroform and ethyl acetate) of *Tamarindus indica* L.. Saudi J Biol Sci..

[84] Simpson BB (1991). The past and present uses of rhatany (*Krameria*, Krameriaceae). Econ Bot.

[85] Scholz E, Rimpler H (1989). Proanthocyanidins from *Krameria triandra* Root. Planta Med.

[86] Baumgartner L, Schwaiger S, Stuppner H (2011). Quantitative analysis of anti-inflammatory lignan derivatives in Ratanhiae radix and its tincture by HPLC–PDA and HPLC–MS. J Pharmaceut Biomed.

[87] Al-Oqail MM (2021). Anticancer efficacies of *Krameria lappacea* extracts against human breast cancer cell line (MCF-7): Role of oxidative stress and ROS generation. Saudi Pharm J.

[88] Alonso-Castro AJ, Zavala-Sánchez MA, Pérez-Ramos J, Sánchez-Mendoza E, Pérez-Gutiérrez S (2015). Antinociceptive and anti-arthritic effects of kramecyne. Life Sci.

[89] Leporatti ML, Pintore G, Foddai M, Chessa M, Piana A, Petretto GL, Masia MD, Mangano G, Nicoletti M (2013). Chemical, biological, morphoanatomical and antimicrobial study of *Ocotea puchury-major* Mart. Nat Prod Res.

[90] Yamaguchi MU, Garcia FP, Cortez DAG, Ueda-Nakamura T, Filho BPD, Nakamura CV (2010). Antifungal effects of Ellagitannin isolated from leaves of *Ocotea odorifera* (Lauraceae). A Van Leeuw J Microb.

[91] Ballabeni V, Tognolini M, Bertoni S, Bruni R, Guerrini A, Rueda GM, Barocelli E (2007). Antiplatelet and antithrombotic activities of essential oil from wild *Ocotea quixos* (Lam.) Kosterm. (Lauraceae) calices from Amazonian Ecuador. Pharmacol Res.

[92] Pimentel EF, Gómes B, Cláudia A, Portes D, Moreira MF, Scherer R, Ruas FG, Romão W, Fronza M, Endringer DC (2024). Polyphenols, antioxidants, and wound healing of *Lecythis pisonis* seed coats. Planta Med.

[93] Kerdel-Vegas F (1966). The depilatory and cytotoxic action of “coco de mono” (*Lecythis ollaria*) and its relationship to chronic seleniosis. Econ Bot.

[94] Dieter M, Desel H (2010). Acute selenium poisoning by paradise nuts (*Lecythis ollaria*). Hum Exp Toxicol.

[95] Araújo SA, Martins A, Silva CR, Bezerra E, Quintino C, Ferreira S, Perales J, Costa-Júnior LM (2017). *In vitro* anthelmintic effects of *Spigelia anthelmia* protein fractions against *Haemonchus contortus*. PLoS ONE.

[96] Vita GF, Ferreira I, Pereira MAVC, Sanavria A, Aurnheimer RCM (2019). Atividade anti-helmíntica de *Spigelia anthelmia* no controle de parasitos gastrintestinais de *Gallus gallus*. Scientia Plena..

[97] Achenbach H, Hübner H, Vierling W, Brandt W, Reiter M (1995). Spiganthine, the cardioactive principle of *Spigelia anthelmia*. J Nat Prod.

[98] Hübner H, Vierling W, Brandt W, Reiter M, Achenbach H (2001). Minor constituents of *Spigelia anthelmia* and their cardiac activities. Phytochemistry.

[99] Camurça-Vasconcelos AL, Nascimento NR, Sousa CM, Melo LM, Morais SM, Bevilaqua CM, Rocha MF (2004). Neuromuscular effects and acute toxicity of an ethyl acetate extract of *Spigelia anthelmia* Linn. J Ethnopharmacol.

[100] Cádiz-Gurrea L, Micol V, Joven J, Segura-Carretero A, Fernández-Arroyo S (2019). Different behavior of polyphenols in energy metabolism of lipopolysaccharide-stimulated cells. Food Res Int.

[101] Nwonuma CO, Abdurrahman JE, Rotimi DE, Evbuomwan IO, Lele KC, Alejolowo OO, Ezea SC, Asogwa NT, Oludipe EO (2023). *Theobroma cacao* fortified-feed ameliorates potassium bromate-induced oxidative damage in male wistar rat. Toxicol Rep.

[102] Ramiro E, Franch À, Castellote C, Pérez-Cano F, Permanyer J, Izquierdo-Pulido M, Castell M (2005). Flavonoids from *Theobroma cacao* down-regulate inflammatory mediators. J Agr Food Chem.

[103] Indla E, Rajasekar K, Kumar B, Kumar S, Chelli S, Sayana SB (2023). Neurohistopathological alterations induced by *Theobroma Cacao* and *Camellia Sinensis* extracts in diabetic male Wistar rats. Cureus.

[104] Patil PP, Khanal P, Patil VS, Charla R, Harish DR, Patil BM, Roy S (2022). Effect of *Theobroma cacao* L. on the efficacy and toxicity of doxorubicin in mice bearing ehrlich ascites carcinoma. Antioxidants.

[105] Mustarichie R, Hasanah AN, Wilar G, Gozali D, Saptarini NM (2022). New hair growth cream formulation with cocoa pod peel (*Theobroma cacao* L.). Sci World J.

[106] Owusu DA, Afedzi AEK, Quansah L (2021). Phytochemical and proximate content of *Carapa procera* bark and its antimicrobial potential against selected pathogens. PLoS ONE.

[107] Benjamin Koama K, Serge Yerbanga R, Roland Meda NT, Ouedraogo N, Da O, Bosco Ouedraogo J, Traore Coulibaly M, Anicet Ouedraogo G (2021). In vivo antimalarial, antioxidant activities and safety of *Carapa procera* DC. (Meliaceae). Pak J Biol Sci.

[108] Mahama A, Chama MA, Oppong Bekoe E, Asare GA, Obeng-Kyeremeh R, Amoah D, Agbemelo-Tsomafo C, Amoah LE, Erskine IJ, Kusi KA, Adjei S (2022). Assessment of toxicity and anti-plasmodial activities of chloroform fractions of *Carapa procera* and *Alchornea cordifolia* in murine models. Front Pharmacol.

[109] MacKinnon S, Durst T, Arnason JT, Angerhofer C, Pezzuto J, Sanchez-Vindas PE, Poveda LJ, Gbeassor M (1997). Antimalarial activity of tropical Meliaceae extracts and gedunin derivatives. J Nat Prod.

[110] Omar S, Godard K, Ingham A, Hussain H, Wongpanich V, Pezzuto J, Durst T, Eklu C, Gbéassor M, Sánchez-Vindas P, Poveda L, Philogene BJR, Arnason JT (2003). Antimalarial activities of gedunin and 7-methoxygedunin and synergistic activity with dillapiol. Ann Appl Biol.

[111] González-Coloma A, Reina M, Sáenz C, Lacret R, Ruiz-Mesia L, Arán VJ, Sanz J, Martínez-Díaz RA (2011). Antileishmanial, antitrypanosomal, and cytotoxic screening of ethnopharmacologically selected Peruvian plants. Parasitol Res.

[112] Lemus de la Cruz AS, Barrera-Cortés J, Lina-García LP, Ramos-Valdivia AC, Santillán R (2022). Nanoemulsified formulation of *Cedrela odorata* essential oil and its larvicidal effect against *Spodoptera frugiperda* (J.E. Smith). Molecules.

[113] Giordani MA, Collicchio TC, Ascêncio SD, Martins DT, Balogun SO, Bieski IG, da Silva LA, Colodel EM, de Souza RL, de Souza DL, de França SA, Andrade CM, Kawashita NH (2015). Hydroethanolic extract of the inner stem bark of *Cedrela odorata* has low toxicity and reduces hyperglycemia induced by an overload of sucrose and glucose. J Ethnopharmacol.

[114] Lee HS, Park JW, Kwon OK, Lim Y, Kim JH, Kim SY, Zamora N, Rosales K, Choi S, Oh SR, Ahn KS (2019). Anti-inflammatory effects of ethanol extract from the leaves and shoots of *Cedrela odorata* L. in cytokine-stimulated keratinocytes. Exp Ther Med.

[115] Phillips O (1990). *Ficus insipida* (Moraceae): ethnobotany and ecology of an Amazonian anthelmintic. Econ Bot.

[116] Stepek G, Lowe AE, Buttle DJ, Duce IR, Behnke JM (2007). *In vitro* anthelmintic effects of cysteine proteinases from plants against intestinal helminths of rodents. J Helminthol.

[117] Pereira ACH, Lenz D, Nogueira BV, Scherer R, Andrade TU, Costa HB, Romão W, Pereira TMC, Endringer DC (2017). Gastroprotective activity of the resin from *Virola oleifera*. Pharm Biol.

[118] Roumy V, Celidonio J, Bonneau N, Samaillie J, Azaroual N, Encinas LA, Rivière C, Hennebelle T, Sahpaz S, Anthérieu S, Pinçon C, Neut C, Siah A, Gutierrez-Choquevilca AL, Ruiz L (2020). Plant therapy in the Peruvian Amazon (Loreto) in case of infectious diseases and its antimicrobial evaluation. J Ethnopharmacol.

[119] Carvalho GR, Braz DS, Gonçalves TCO, Aires R, Côco LZ, Guidoni M, Fronza M, Endringer DC, Júnior ADS, Campos-Toimil M, Nogueira BV, Vasquez EC, Campagnaro BP, Pereira TMC (2022). Development and evaluation of *Virola oleifera* formulation for cutaneous wound healing. Antioxidants (Basel).

[120] Carvalho AA, Galdino PM, Nascimento MV, Kato MJ, Valadares MC, Cunha LC, Costa EA (2010). Antinociceptive and antiinflammatory activities of grandisin extracted from *Virola surinamensis*. Phytother Res.

[121] Lopes NP, Chicaro P, Kato MJ, Albuquerque S, Yoshida M (1998). Flavonoids and Lignans from *Virola surinamensis* twigs and their *in vitro* activity against *Trypanosoma cruzi*. Planta Med.

[122] Barata LES, Santos LS, Ferri PH, Phillipson JD, Paine A, Croft SL (2000). Anti-leishmanial activity of neolignans from *Virola* species and synthetic analogues. Phytochemistry.

[123] Paes SS, Silva-Silva JV, Gomes PWP, Silva LO, Costa APL, Júnior MLL, Hardoim DJ, Moragas-Tellis CJ, Taniwaki NN, Bertho AL, Molfetta FA, Almeida-Souza F, Santos LS, Calabrese KS (2023). (-)-5-Demethoxygrandisin B, a new lignan from *Virola surinamensis* (Rol.) Warb. leaves: evaluation of the leishmanicidal activity by in vitro and in silico approaches. Pharmaceutics.

[124] González-Rodríguez M, Ruiz-Fernández C, Francisco V, Ait Eldjoudi D, Farrag AbdElHafez YR, Cordero-Barreal A, Pino J, Lago F, Campos-Toimil M, Rocha Carvalho G, Melo Costa Pereira T, Gualillo O (2021). Pharmacological extracts and molecules from *Virola* Species: traditional uses, phytochemistry, and biological activity. Molecules.

[125] Basting RT, Nishijima CM, Lopes JA, Santos RC, Lucena LP, Laufer S, Bauer S, Costa MF, Santos LC, Rocha LRM, Vilegas W, Santos ARS, Santos C, Hiruma-Lima CA (2014). Antinociceptive, anti-inflammatory and gastroprotective effects of a hydroalcoholic extract from the leaves of *Eugenia punicifolia* (Kunth) DC. in rodents. J Ethnopharmacol.

[126] Périco LL, Rodrigues VP, Ohara R, Nunes VVA, Rocha LRM, Vilegas W, Santos C, Hiruma-Lima CA (2019). Can the gastric healing effect of Eugenia punicifolia be the same in male and female rats?. J Ethnopharmacol.

[127] Silva SM, Costa CRR, Martins Gelfuso GM, Guerra ENS, Nóbrega YKM, Gomes SM, Pic-Taylor A, Fonseca-Bazzo YM, Silveira D, Magalhães PO (2018). Wound healing effect of essential oil extracted from *Eugenia dysenterica* DC (Myrtaceae) leaves. Molecules.

[128] Birdi T, Daswani P, Brijesh S, Tetali P, Natu A, Antia N (2010). Newer insights into the mechanism of action of *Psidium guajava* L. leaves in infectious diarrhea. BMC Comp Altern Med..

[129] Morais-Braga MF, Carneiro JN, Machado AJ, Dos Santos AT, Sales DL, Lima LF, Figueredo FG, Coutinho HD (2016). *Psidium guajava* L., from ethnobiology to scientific evaluation: Elucidating bioactivity against pathogenic microorganisms. J Ethnopharmacol.

[130] Jamieson S, Wallace CE, Das N, Bhattacharyya P, Bishayee A (2023). Guava (*Psidium guajava* L.): a glorious plant with cancer preventive and therapeutic potential. Crit Rev Food Sci Nutr.

[131] Gunn JA (1927). The action of expectorants. Brit Med J.

[132] Robertson WO (1962). Syrup of ipecac: A slow or fast emetic?. Am J Dis Child.

[133] Saetta JP, Quinton DN (1991). Residual gastric content after gastric lavage and ipecacuanha-induced emesis in self-poisoned patients: an endoscopic study. J R Soc Med.

[134] Falanga CM, Steinborn C, Muratspahić E, Zimmermann-Klemd AM, Winker M, Krenn L, Huber R, Gruber CW, Gründemann C (2022). Ipecac root extracts and isolated circular peptides differentially suppress inflammatory immune response characterised by proliferation, activation and degranulation capacity of human lymphocytes in vitro. Biomed Pharmacother.

[135] Araujo DF, Holanda BF, Nascimento FLFD, Martins AB, Silva ALM, Pereira MG, Freitas Pires A, Assreuy AMS (2024). Polysaccharide-rich extract of *Genipa americana* leaves exerts anti-inflammatory effects modulated by platelet mediators. J Ethnopharmacol.

[136] Neves MIL, Socas-Rodríguez B, Valdés A, Silva EK, Cifuentes A, Meireles MAA, Ibáñez E (2022). Synergic effect of natural deep eutectic solvent and high-intensity ultrasound on obtaining a ready-to-use genipin extract: crosslinking and anti-neurodegenerative properties. Food Chem.

[137] Santos AC, Otsuka FAM, Santos RB, de Trindade DJ, Matos HR (2022). Antiglycation potential and antioxidant activity of genipap (*Genipa americana* L.) in oxidative stress mediated by hydrogen peroxide on cell culture. Nat Prod Res.

[138] Madeira JC, Farias LAS, Luz CP, Assreuy AMS, Pereira MG (2020). Per oral rat treatment with glyconjugate fractions of *Genipa americana* leaves protects thrombus formation. Blood Coagul Fibrin.

[139] Nonato DTT, Vasconcelos SMM, Mota MRL, de Silva PGB, Cunha AP, Ricardo NMPS, Pereira MG, Assreuy AMS, Chaves EMC (2018). The anticonvulsant effect of a polysaccharide-rich extract from *Genipa americana* leaves is mediated by GABA receptor. Biomed Pharmacother.

[140] da Souza ROS, Sousa PL, de Menezes RRPPB, Sampaio TL, Tessarolo LD, Silva FCO, Pereira MG, Martins AMC (2018). Trypanocidal activity of polysaccharide extract from *Genipa americana* leaves. J Ethnopharmacol.

[141] Espinola EB, Dias RF, Mattei R, Carlini EA (1997). Pharmacological activity of Guarana (*Paullinia cupana* Mart.) in laboratory animals. J Ethnopharmacol.

[142] Haskell CF, Kennedy DO, Wesnes KA, Milne AL, Scholey AB (2006). A double-blind, placebo-controlled, multi-dose evaluation of the acute behavioural effects of guaraná in humans. J Psychopharmacol.

[143] Campos MPO, Riechelmann R, Martins LC, Hassan BJ, Casa FBA, Giglio AD (2011). Guarana (*Paullinia cupana*) improves fatigue in breast cancer patients undergoing systemic chemotherapy. J Altern Complement Med.

[144] Lima NDS, Numata EP, Mesquita LMS, Dias PH, Vilegas W, Gambero A, Ribeiro ML (2017). Modulatory effects of Guarana (*Paullinia cupana*) on adipogenesis. Nutrients.

[145] Lima NDS, Caria CREP, Gambero A, Ribeiro ML (2019). The effect of Guarana (*Paullinia cupana*) on metabolic and inflammatory parameters in adult male mice programmed by maternal obesity. Eur J Nutr.

[146] Teixeira CD, Barbosa PO, Souza MO (2024). Effects of guarana (*Paullinia cupana*) powder on obesity-associated diseases in animal models: a systematic review. J Funct Foods.

[147] Ruchel JB, Bernardes VM, Braun JBS, Manzoni AG, Passos DF, Castilhos LG, Abdalla FH, de Oliveira JS, de Andrade CM, Casali EA, da Cruz IBM, Leal DBR (2021). Lipotoxicity-associated inflammation is prevented by guarana (*Paullinia cupana*) in a model of hyperlipidemia. Drug Chem Toxicol.

[148] Lorz L, Yoo B, Kim M-Y, Cho J (2019). Anti-wrinkling and anti-melanogenic effect of *Pradosia mutisii* methanol extract. Int J Mol Sci.

[149] Abdala S, Martín-Herrera D, Benjumea D, Gutiérrez SD (2012). Diuretic activity of some *Smilax canariensis* fractions. J Ethnopharmacol.

[150] Abdala S, Martín-Herrera D, Benjumea D, Pérez-Paz P (2008). Diuretic activity of *Smilax canariensis*, an endemic Canary Island species. J Ethnopharmacol.

[151] Pereira FL, Oliveira VB, Viana CTR, Campos PP, Silva MAN, Brandão MGL (2015). Antihyperlipidemic and antihyperglycemic effects of the Brazilian salsaparrilhas *Smilax brasiliensis* Spreng. (Smilacaceae) and *Herreria salsaparrilha* Mart. (Agavaceae) in mice treated with a high-refined-carbohydrate containing diet. Food Res Int.

[152] Romo-Pérez A, Escandón-Rivera SM, Andrade-Cetto A (2019). Chronic hypoglycemic effect and phytochemical composition of Smilax moranensis roots. Rev Bras Farmacogn.

[153] Khan AK, Singh PD, Reese PB, Howden J, Thomas TT (2019). Investigation of the anti-inflammatory and the analgesic effects of the extracts from *Smilax ornata* Lem. (Jamaican sarsaparilla) plant. J Ethnopharmacol.

[154] Oliveira JE, Romero MA, Silva MS, Silva BA, Medeiros IA (2001). Intracellular calcium mobilization as a target for the spasmolytic action of scopoletin. Planta Med.

[155] Weimer P, Spies LM, Haubert R, de Lima JAS, Maluf RW, Rossi RC, Suyenaga ES (2021). Anti-inflammatory activity of *Brunfelsia uniflora* root extract: phytochemical characterization and pharmacologic potential of this under-investigated species. Nat Prod Res.

[156] Sugauara EYY, Sugauara E, Sugauara RR, Bortolucci WC, Fernandez CMM, Gonçalves JE, Colauto NB, Gazim ZC, Linde GA (2022). Larvicidal activity of *Brunfelsia uniflora* extracts on *Aedes aegypti* larvae. Nat Prod Res.

[157] Thiesen LC, Sugauara EY, Tešević V, Glamočlija J, Soković M, Gonçalves JE, Gazim ZC, Linde GA, Colauto NB (2017). Antimicrobial activity and chemical composition of *Brunfelsia uniflora* flower oleoresin extracted by supercritical carbon dioxide. Genet Mol Res.

[158] Rodrigues FAP, Prata MMG, Oliveira ICM, Alves NTQ, Freitas REM, Monteiro HSA, Silva JA, Vieira PC, Viana DA, Libório AB, Havt A (2014). Gingerol fraction from *Zingiber officinale* protects against gentamicin-induced nephrotoxicity. Antimicrob Agents Chemother.

[159] Shimoda H, Shan S-J, Tanaka J, Seki A, Seo J-W, Kasajima N, Tamura S, Ke Y, Murakami N (2010). Anti-inflammatory properties of red ginger (*Zingiber officinale* var. *rubra*) extract and suppression of nitric oxide production by its constituents. J Med Food.

[160] Ansari JA, Ahmad MK, Khan AR, Fatima N, Khan HJ, Rastogi N, Mishra DP, Mahdi AA (2016). Anticancer and antioxidant activity of *Zingiber officinale* Roscoe rhizome. Indian J Exp Biol.

[161] Fajrin FA, Imandasari N, Barki T, Sulistyaningrum G, Kristiningrum N, Puspitasari E, Holidah D (2019). The activity of red ginger oil in antioxidant study in vitro and antihyperalgesia effect in alloxan- induced painful diabetic neuropathy in mice. Thai J Pharm Sci.

[162] Yocum GT, Hwang JJ, Mikami M, Danielsson J, Kuforiji AS, Emala CW (2020). Ginger and its bioactive component 6-shogaol mitigate lung inflammation in a murine asthma model. Am J Physiol-Lung C.

[163] Pironon S, Ondo I, Diazgranados M, Allkin R, Baquero AC, Cámara-Leret R, Canteiro C, Dennehy-Carr Z, Govaerts R, Hargreaves S, Hudson AJ, Lemmens R, Milliken W, Nesbitt M, Patmore K, Schmelzer G, Turner RM, van Andel TR, Ulian T, Antonelli A, Willis KJ (2024). The global distribution of plants used by humans. Science.

[164] Bennett BC (1992). Plants and People of the Amazonian Rainforests. Bioscience.

[165] Albuquerque UP, Hanazaki N (2006). As pesquisas etnodirigidas na descoberta de novos fármacos de interesse médico e farmacêutico: fragilidades e perspectivas. Rev Bras Farmacogn.

[166] Silva TC, Medeiros PM, Balcazár AL, Araújo TAS, Pirondo A, Medeiros MFT (2014). Historical ethnobotany: an overview of selected studies. Ethnobiol Conserv..

[167] Odonne G, Tareau MA, van Andel T (2021). Geopolitics of bitterness: Deciphering the history and cultural biogeography of *Quassia amara* L. J Ethnopharmacol.

[168] Rocha LPB, Alves JVO, Aguiar IFS, Silva FH, Silva RL, Arruda LG, Nascimento Filho EJ, Barbosa BVD, Amorim LC, Silva PM, Silva MV (2021). Use of medicinal plants: History and relevance. Res Soc Dev..

[169] Santa-Anna Nery JF (1889). Folk-lore: poésie populaire, contes et légendes, fables et mythes, poésie, musique, danses et croyances des indiens.

[170] Sallas ALF. Ciência do homem e sentimento da natureza: viajantes alemães no Brasil do século XIX. Curitiba: Editora da UFPR; 2013.

[171] Santos FSD (2000). Tradições populares de uso de plantas medicinais na Amazônia. Hist Cienc Saude-Manguinhos.

[172] Larrea-Alcázar DM, Cuvi N, Valentim JF, Diaz L, Vidal S, Palacio G. Economic drivers in the Amazon from the 19th century to the 1970s. In: Nobre C, et al., editors. Science Panel for the Amazon. Executive Summary of the Amazon Assessment Report 2021. New York: United Nations Sustainable Development Solutions Network; 2021. p. 1–25.

[173] Schultes RE (1990). Gifts of the Amazon Flora to the World. Arnoldia.

[174] Alcantara-Rodriguez M, Françozo M, van Andel T (2019). Plant Knowledge in the *Historia Naturalis Brasiliae* (1648): Retentions of Seventeenth-Century Plant Use in Brazil. Econ Bot.

[175] Tabajara de Oliveira Martins D, Rodrigues E, Casu L, Benítez G, Leonti M (2019). The historical development of pharmacopoeias and the inclusion of exotic herbal drugs with a focus on Europe and Brazil. J Ethnopharmacol.

[176] Bieski IG, Leonti M, Arnason JT, Ferrier J, Rapinski M, Violante IM, Balogun SO, Pereira JF, Figueiredo Rde C, Lopes CR, da Silva DR, Pacini A, Albuquerque UP, Martins DT (2015). Ethnobotanical study of medicinal plants by population of Valley of Juruena Region, Legal Amazon, Mato Grosso. Brazil J Ethnopharmacol.

[177] Pedrollo CT, Kinupp VF, Shepard G, Heinrich M (2016). Medicinal plants at Rio Jauaperi, Brazilian Amazon: Ethnobotanical survey and environmental conservation. J Ethnopharmacol.

[178] Levis C, Flores BM, Moreira PA, Luize BG, Alves RP, Franco-Moraes J, Lins J, Konings E, Peña-Claros M, Bongers F, Costa FRC, Clement CR (2018). How People Domesticated Amazonian Forests. Front Ecol Evol.

[179] Santos RS, Coelho-Ferreira M, Lima PGC, Magalhães MP (2019). Useful plants and their relation to archaeological sites in the Serra de Carajás. Brazil An Acad Bras Cienc.

[180] Geertsma IP, Françozo M, van Andel T, Rodríguez MA (2021). What's in a name? Revisiting medicinal and religious plants at an Amazonian market. J Ethnobiol Ethnomed.

[181] Oliveira Melo PMC, Lima PGC, Costa JC, Coelho-Ferreira MR (2022). Ethnobotanical study in a rural settlement in Amazon: contribution of local knowledge to public health policies. Res Soc Dev..

[182] Sõukand R, Kalle R, Prakofjewa J, Sartori M, Pieroni A (2024). The importance of the continuity of practice: Ethnobotany of Kihnu island (Estonia) from 1937 to 2021. Plants People Planet..

[183] Mugge FLB, Baratto LC, Brandão MGL, Oliveira WP (2022). Importance of Historical Records for the Development of Herbal Medicines: The Example of COVID-19. Phytotechnology: A Sustainable Platform for the Development of Herbal Products.

[184] Mügge FLB, Paula-Souza J, Melo JC, Brandão MGL (2016). Native plant species with economic value from Minas Gerais and Goiás: a discussion on the currentness of the data recovered by the French naturalist Auguste de Saint-Hilaire. Hortic Bras.

[185] Baratto LC. O conhecimento tradicional associado à biodiversidade amazônica e o potencial bioeconômico da floresta. In: Almeida AEM, Oliveira EG, Abreu VHR, Baratto LC, Nunes KM, editors. Manual fitoterápico amazônico com foco na atenção Básica sob a ótica da interdisciplinaridade. Macapá: UNIFAP; 2023.p. 11–20. http://www2.unifap.br/editora/files/2023/04/MANUAL-FITOTERAPICO-AMAZONICO-COM-FOCO-NA-ATENCAO-BASICA-SOB-A-OTICA-DA-INTERDISCIPLINARIDADE.pdf

[186] Rivadeneyra-Domínguez E, Rodríguez-Landa JF (2020). Preclinical and clinical research on the toxic and neurological effects of cassava (*Manihot esculenta* Crantz) consumption. Metab Brain Dis.

[187] Teixeira JRM, Lapa AJ, Souccar C, Valle JR (1984). Timbós: ichthyotoxic plants used by Brazilian indians. J Ethnopharmacol.

[188] Muñoz V, Sauvain M, Bourdy G, Callapa J, Bergeron S, Rojas I, Bravo JA, Balderrama L, Ortiz B, Gimenez A, Deharo E (2000). A search for natural bioactive compounds in Bolivia through a multidisciplinary approach: Part I: Evaluation of the antimalarial activity of plants used by the Chacobo Indians. J Ethnopharmacol.

[189] Carod-Artal FJ, Vázquez-Cabrera CB (2007). An Anthropological Study about Epilepsy in Native Tribes from Central and South America. Epilepsia.

[190] Díaz-Reviriego I, Fernández-Llamazares A, Howard PL, Molina JL, Reyes-García V (2016). Fishing in the Amazonian forest: a gendered social network puzzle. Soc Natl Resources..

[191] Pontes CJF (2015). A guerra no inferno verde: Segundo Ciclo da borracha, o front da Amazônia e os Soldados da Borracha. South Am J Basic Educ Techn Technol.

[192] Pontes CJF (2014). O primeiro ciclo da borracha no acre: da formação dos seringais ao grande colapso. South Am J Basic Educ Techn Technol.

[193] Dutra RC, Campos MM, Santos AR, Calixto JB (2016). Medicinal plants in Brazil: Pharmacological studies, drug discovery, challenges and perspectives. Pharmacol Res.

[194] Walker TH, Chaar JM, Mehr CB, Collins JL, Parliament TH, Ho CT, Schieberle P (2000). The Chemistry of Guaraná: Guaraná, Brazil’s Super-Fruit for the Caffeinated Beverages Industry. Caffeinated Beverages: Caffeinated Beverages: Health Benefits, Physiological Effects, and Chemistry.

[195] Schimpl FC, da Silva JF, Gonçalves JF, Mazzafera P (2013). Guarana: revisiting a highly caffeinated plant from the Amazon. J Ethnopharmacol.

[196] Atanasov GA, Waltenberger B, Pferschy-Wenzig EM, Linder T, Wawrosch C, Uhrin P, Temml V, Wang L, Schwaiger S, Heiss EH, Rollinger JM, Schuster D, Breuss JM, Bochkov V, Mihovilovic MD, Kopp B, Bauer R, Dirsch VM, Stuppner H (2015). Discovery and resupply of pharmacologically active plant-derived natural products: a review. Biotechnol Adv.

[197] Lucena CM, Lucena RFP, Lucena RFP, Albuquerque UP, Lucena CM, Ferreira EC (2020). Histórico, definição e importância da Etnobotânica. Perspectivas e avanços na etnobiologia: uma avaliação na Conferência Internacional do Brasil.

[198] Cardoso D, Särkinen T, Alexander SN, Amorim AM, Bittrich V, Celis M, Forzza RC (2017). Amazon plant diversity revealed by a taxonomically verified species list. Proc Natl Acad Sci.

[199] Flora e Funga do Brasil. Jardim Botânico do Rio de Janeiro. http://floradobrasil.jbrj.gov.br/. Accessed 14 Dec 2023.

[200] Hubbell SP, He F, Condit R, Borda-de-Água L, Kellner JR, Steege HT (2008). How many tree species are there in the amazon and how many of them will go extinct?. Proc Natl Acad Sci.

